# Enhanced cortical neural stem cell identity through short SMAD and WNT inhibition in human cerebral organoids facilitates emergence of outer radial glial cells

**DOI:** 10.1038/s41556-022-00929-5

**Published:** 2022-06-13

**Authors:** Daniel Rosebrock, Sneha Arora, Naresh Mutukula, Rotem Volkman, Elzbieta Gralinska, Anastasios Balaskas, Amèlia Aragonés Hernández, René Buschow, Björn Brändl, Franz-Josef Müller, Peter F. Arndt, Martin Vingron, Yechiel Elkabetz

**Affiliations:** 1 Department of Genome Regulation, Max Planck Institute for Molecular Genetics, Berlin, Germany; 2 Department of Computational Biology, Max Planck Institute for Molecular Genetics, Berlin, Germany; 3 Department of Mathematics and Computer Science, Freie Universität Berlin, Berlin, Germany; 4 Department of Cell and Developmental Biology, Sackler School of Medicine, Tel Aviv University, Tel Aviv, Israel; 5 Institute of Biology, Department of Biology, Chemistry, and Pharmacy, Freie Universität Berlin, Berlin, Germany; 6 Institute of Chemistry and Biochemistry, Department of Biology, Chemistry and Pharmacy, Freie Universität Berlin, Berlin, Germany; 7 Microscopy and Cryo-Electron Microscopy, Max Planck Institute for Molecular Genetics, Berlin, Germany; 8 Department of Psychiatry and Psychotherapy, University Hospital Schleswig Holstein, Kiel, Germany

**Keywords:** Developmental neurogenesis, Neural stem cells

## Abstract

Cerebral organoids exhibit broad regional heterogeneity accompanied by limited cortical cellular diversity despite the tremendous upsurge in derivation methods, suggesting inadequate patterning of early neural stem cells (NSCs). Here we show that a short and early Dual SMAD and WNT inhibition course is necessary and sufficient to establish robust and lasting cortical organoid NSC identity, efficiently suppressing non-cortical NSC fates, while other widely used methods are inconsistent in their cortical NSC-specification capacity. Accordingly, this method selectively enriches for outer radial glia NSCs, which cyto-architecturally demarcate well-defined outer sub-ventricular-like regions propagating from superiorly radially organized, apical cortical rosette NSCs. Finally, this method culminates in the emergence of molecularly distinct deep and upper cortical layer neurons, and reliably uncovers cortex-specific microcephaly defects. Thus, a short SMAD and WNT inhibition is critical for establishing a rich cortical cell repertoire that enables mirroring of fundamental molecular and cyto-architectural features of cortical development and meaningful disease modelling.

## Abstract (web summary)

Rosebrock, Arora et al. report a method to overcome limited cortical cellular diversity in human organoids, thus mirroring fundamental features of cortical development and offering a basis for organoid-based disease modelling.

## Main

Correct development and expansion of diverse neural cell types in the cerebral cortex relies on the ability of early cortical neuroepithelial cells and radial glial cells—the starting neural stem cell (NSC) population of cortical development^1^—to maintain adequate levels of self-renewal and differentiation capacities. Deviations from this highly ordered process, often associated with pathological conditions or evolutional changes, entail inherent changes in cortical progenitor cell biology^2^. Therefore, the development of gold-standard in vitro strategies for generating precise, stage-matched, homogeneous cortical cell types across various pluripotent stem cell (PSC) sources is fundamental for comparative studies of development, disease and evolution.

The advent of PSCs led to the establishment of various methods for deriving cortical fates. However, protocols are highly diverse and rapidly expanding. Pioneering work on two-dimensional (2D) systems launched by the Sasai group utilized NODAL- and WNT-pathway antagonists for derivation of general telencephalic fates from mouse PSCs^3^. We later recapitulated this default neural induction mechanism in human PSCs and using the BMP antagonist NOG (Noggin), we isolated neural rosette-forming NSCs corresponding to early anterior radial glial-like NSCs^4,5^. This method was substantially improved by adding TGFB inhibition to BMP inhibition, becoming the widely accepted ‘Dual SMAD inhibition protocol’ (Dual SMAD-i) in human PSCs^6^. We previously utilized this method to derive consecutive cortical NSC stages from human PSCs, but added a purification step by isolating NOTCH-active rosettes as a readout for cortical NSC identity^7^. Additional studies employed combinations of TGFB-, BMP- and WNT-pathway inhibition with or without FGF- or SHH-pathway modulation^8–16^. Some of these studies combined WNT inhibition (WNT-i) with Dual SMAD-i. One study for example implicated the type of WNT-inhibitor in cortical neuronal subtype outcome^12^, whereas another study combined WNT, FGF and NOTCH inhibition to induce rapid production of early cortical neurons^13^. A third study suggested the addition of WNT-i on top of Dual SMAD-i only as an optional step^16^. These studies exemplify the heterogeneity across approaches in recapitulating cortical development.

Parallel to 2D differentiation systems, there has been a rapid expansion in the development and utilization of cerebral organoid models, enabling three-dimensional (3D) in vivo-like views of fundamental neurodevelopmental features of corticogenesis in health and disease. Nonetheless, the methods used for generating cortical organoid fates are also highly variable, ranging from inhibitor-free conditions^17–19^ to Dual SMAD-i^20–22^, TGFB and WNT inhibition^23–28^ and combined Dual SMAD-i and WNT-i (triple inhibition, Triple-i)^29^.

One striking landmark of the transition from 2D to 3D systems is the collective agreement that organoids self-form, express general neural/cortical marker genes and exhibit cyto-architectural features regardless of the derivation method used. However, different methods using diverse inhibitory arms could also lead to differential neural patterning trajectories^30^ and consequently to differential cellular identity composition. Furthermore, the lack of specific markers that unequivocally distinguish cortical from non-cortical cell populations, the use of late-appearing cortical markers as gold-standard probes to assess differentiation success and the fact that various methods are not run in parallel in the same study all further confound the validity of measured phenotypes. One recent study by Kriegstein and colleagues compared organoids derived by commonly used methods with in vivo cortical tissue datasets to highlight imperfections in recapitulating distinct developmental cellular identities in vitro regardless of the derivation method^31^. Together, these ideas raise the fundamental question of whether the general lack of standardization in the field adversely affects the interpretation of disease-model phenotypes and their implication in regenerative medicine.

We reasoned that heterogeneity within and among organoids reflects inefficient patterning of early organoid NSCs. We generated organoids and compared standard and more directed derivation methods side by side at the transcriptional, cellular and cyto-architectural level, with particular focus on the regional composition of NSCs. Through integration of bulk RNA sequencing (RNA-Seq) and single-cell RNA-Seq (scRNA-Seq) datasets of organoids derived using these methods together with published datasets obtained from human brain samples, we pinpointed major differences between NSC regional compositions across methods. Strikingly, we revealed that a short and early exposure to Triple-i inhibition both enriches for cortical NSC identity and suppresses non-cortical NSC fates. We further identified enrichment for outer radial glia (oRG) cells in these organoids. Finally, we show that this method facilitates a robust radial organization of NSCs—the cyto-architectural grounds for the formation of well-defined cortical germinal zones, that is, the ventricular, inner sub-ventricular and outer sub-ventricular zones (VZ, iSVZ and oSVZ, respectively)—and enables a more meaningful modelling of microcephaly model. These findings underscore the indispensable role of our method in establishing a solid molecular and cyto-architectural foundation of cortical NSCs that is required for building a rich cortical organoid cellular diversity and uncovering unique cortex-specific disease aetiologies.

## Results

### Short Triple-i enriches organoid cortical identity

**Extended Data Fig. 1:**
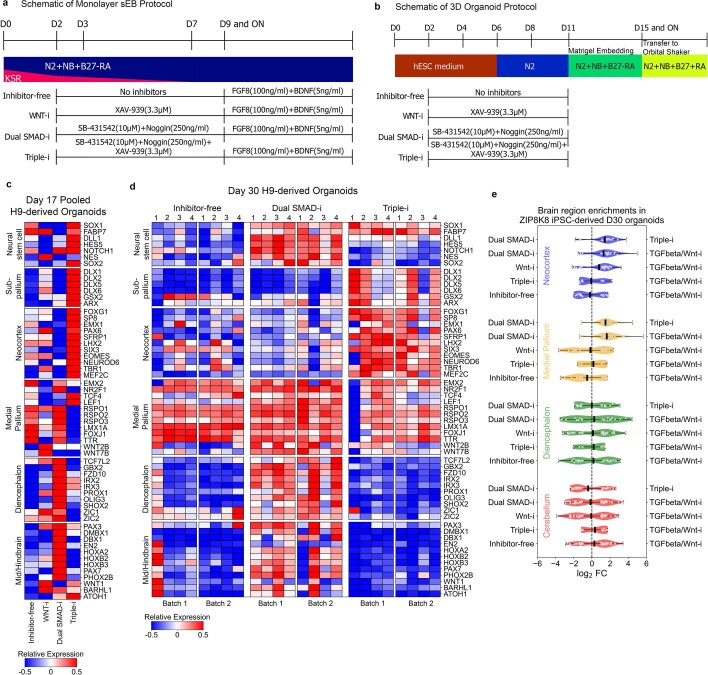
Differentiation Schematics, regional transcriptomic characterization of organoids derived by various methods, and iPSC line characterization. **a**. Schematic representation of 2D monolayer neural rosette differentiation protocol from human PSCs. **b**. Schematic showing generation of 3D cerebral organoids from human PSCs. Note that the factors were added to the culture from day 2 until day 10 of differentiation protocol. **c**. A heatmap representing expression values for selected genes categorized according to neural stem cell markers and additional groups of regional markers (sub-pallium, neocortex, medial pallium, diencephalon and midbrain-hindbrain) in pooled day 17 H9 hESC-derived organoids derived under indicated treatments (4 organoids pooled in each bulk RNA-Seq sample). Colour-coded scale represents relative expression levels of each gene (row) across treatments. **d**. A heatmap representing expression values for selected genes categorized according to neural stem cell markers and additional groups of regional markers (sub-pallium neocortex, medial pallium, diencephalon and midbrain-hindbrain) in individual day 30 H9 hESC-derived organoids derived under indicated treatments and separated by batch (N=8 individual organoids for each protocol collected in groups of four organoids per protocol in 2 batches). Colour-coded scale represents relative expression levels of each gene (row) across treatments. **e**. Violin plot of differentially expressed genes (|log2 fold change| ≥ 1) among ZIP8K8 iPSC derived pooled day 30 organoids (N=1 Dual SMAD-i, 5 organoids pooled; N=1 Inhibitor-free, 5 organoids pooled; N=1 WNT-i, 4 organoids pooled; N=1 TGFB/WNT-i, 4 organoids pooled; N=6 Triple-i, of which 5 organoids pooled (N=1) and 4 organoids pooled (N=5)) compared under indicated treatments.

To dissect the necessity of different inhibition variants used in currently published protocols for achieving cortical fates, we compared organoids generated using the standard inhibition-free protocol^32^ (denoted as Inhibitor-free) with those generated by the WNT inhibitor XAV-939 alone (WNT-i) or the TGFB and BMP inhibitors SB-431542 and NOG combined (Dual SMAD-i)^6^ as controls as well as with organoids made by Triple-i inhibition as the most directed cortical differentiation paradigm^26,33^ (see Extended Data Fig. 1a,b for a detailed schematic).

**Fig. 1:**
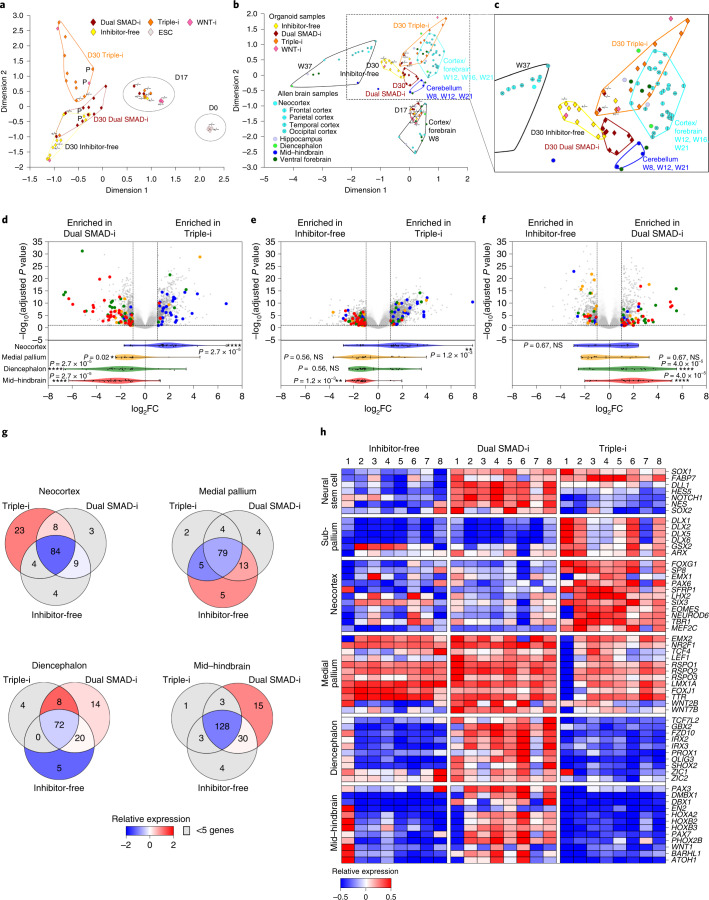
Combined global gene expression analysis of H9 hESC-derived organoids and human brain samples reveal distinct brain region specification by various protocols. **a**, Correspondence analysis plot of RNA-Seq datasets obtained from undifferentiated PSCs and day 17 and 30 organoids derived from the H9 ESC cell line using the indicated treatments. Transcriptome profiles were obtained from single individually processed day 30 organoids (*n* = 2 WNT-i-derived organoids collected in two batches and *n* = 8 for all other protocols, collected in groups of four organoids per protocol in two batches), pool-processed day 17 organoids (*n* = 4 organoids, pooled) and pool-processed day 30 organoids (*n* = 3 organoids, pooled). These microcephaly mutant organoids will be described in a later section of the paper. **b**, Correspondence analysis plots calculated for RNA-Seq datasets obtained from **a** and then integrated with human brain region-specific RNA-Seq datasets (Allen Human Brain Atlas, http://human.brain-map.org/). Brain-region-specific samples are shown as circles. See Methods for further subdivisions according to Šestan and colleagues^34^. **c**, Large-scale cluster distribution of **b**. **a**–**c**, P, pooled organoids; −/− and +/−, homozygous and heterozygous microcephaly mutant organoids, respectively. **d**, Volcano plot of day 30 Triple-i organoids compared with Dual SMAD-i organoids (top). DESeq2 was used to estimate the log_2_-transformed fold-change and adjusted *P* values after Benjamini–Hochberg correction. **e**, Day 30 Triple-i organoids compared with Inhibitor-free organoids. **f**, Day 30 Dual SMAD-i organoids compared with Inhibitor-free organoids. **d**–**f**, Region-specific genes from the Allen Human Brain Atlas that were significant differentially expressed are highlighted in the volcano plots and in the violin plot with whiskers extending to the minimum and maximum values and the mean highlighted, along with adjusted *P* values from a gene-set enrichment analysis statistical test after Benjamini–Hochberg correction for regional gene-set enrichments (bottom). *P < 0.05; **P < 0.01; ****P < 0.0001; and NS, not significant. See Methods for details. **g**, Venn diagrams of protocol-specific differentially expressed and non-differentially expressed genes from Allen Human Brain Atlas regional gene sets. The colour of the Venn diagram sections represents the relative expression levels of these genes compared with other protocol-specific regional genes across Allen Human Brain Atlas samples from weeks 12–21. **h**, Heatmap representing the expression values for selected genes categorized according to NSC and regional markers in individual day 30 organoids derived under the indicated treatments. The colour-coded scale represents relative expression levels of each gene (row) across treatments. D0, D17 and D30, days 0, 17 and 30, respectively; W8, W12, W16, W21 and W37; weeks 8, 12, 16, 21 and 37, respectively.

We employed bulk RNA-Seq of individual day 30 human embryonic stem cell (hESC)-derived organoids generated using these conditions as well as several organoids pooled and analysed together on days 17 and 30. Correspondence analysis confirmed that undifferentiated PSCs and day 17 and day 30 organoids segregated as separate clusters, reflecting transition from pluripotency to early and then later neural stages, whereas the day 30 samples segregated further according to the different inhibition paradigms (Fig. 1a).

We then correlated the transcriptional differences among organoids made using the various methods to regional biases using a comparative analysis with in vivo human brain development. We integrated our organoid transcriptional datasets with those of 16 fetal brain regions obtained from the Allen Human Brain Atlas study^34^. To remain unbiased, we included the entire developmental range of 8–37 gestational weeks (Fig. 1b,c). This analysis showed that day 17 organoids co-clustered with week 8 brain tissues regardless of method, although regional specification was already present (Extended Data Fig. 1c). On the other hand, day 30 organoids clustered with forebrain/cortical embryonic samples, particularly of weeks 12–21, only if derived by Triple-i (Fig. 1b,c), whereas Dual SMAD-i organoids appeared proximal to the cerebellar embryonic samples and Inhibitor-free organoids were less associated with any of the in vivo developmental stages. This provided a strong indication that early exposure (days 2–11 in our protocol) to combined Dual SMAD-i and WNT-i is sufficient to promote forebrain/cortical specification, whereas methods lacking WNT-i are more compatible with posteriorization.

We extracted region-specific genes derived from Allen Human Brain Atlas samples across developmental weeks that had the strongest overlap with day 30 organoids and performed pairwise differential gene expression comparisons among these inhibition paradigms. This analysis confirmed that Triple-i significantly enriched for cortical markers when compared with Dual SMAD-i and Inhibitor-free conditions (Fig. 1d,e). In contrast, Dual SMAD-i significantly enriched for thalamic and cerebellar markers (Fig. 1d,f). In support of these findings, cortex-specific genes consistently upregulated in Triple-i organoids (23/135) were highly expressed in embryonic cortical samples and mid–hindbrain-specific genes upregulated in Dual SMAD-i organoids (15/185) were highly expressed in embryonic cerebellar samples (Fig. 1g).

We then investigated whether heterogeneity was in part due to differences between individual organoids generated under the same method. Individual Triple-i organoids exhibited a strong and homogenous cortical signature alongside an inconsistent subpallial signature (Fig. 1h and Extended Data Fig. 1d), whereas Dual SMAD-i organoids exhibited a weak cortical signature alongside a consistent non-cortical signature. Interestingly, the Inhibitor-free organoids exhibited sporadic expression of neocortical and posterior markers, providing an argument for the inconsistency of this method. Finally, the expression levels of medial pallium-fate marker genes were comparable under all methods, suggesting that the patterning of this conserved hippocampal organizer originating in the cortical hem region^35,36^ is inherently resilient to pathway modulation.

Transcriptomic analysis of day 30 organoids derived from ZIP8K8 induced PSCs (iPSCs) revealed similar treatment-dependent regional signature patterns (Extended Data Fig. 1e). Furthermore, we also generated organoids derived under TGFB and WNT inhibition alone. These organoids enriched well for cortical fates similar to Triple-i organoids, although they did not restrict posterior identity as firmly as those under Triple-i. These findings manifest the established instrumental role of BMP inhibition in the acquisition of anterior fates^37^.

### NOTCH activity and radial organization hallmark cortical NSCs

**Extended Data Fig. 2:**
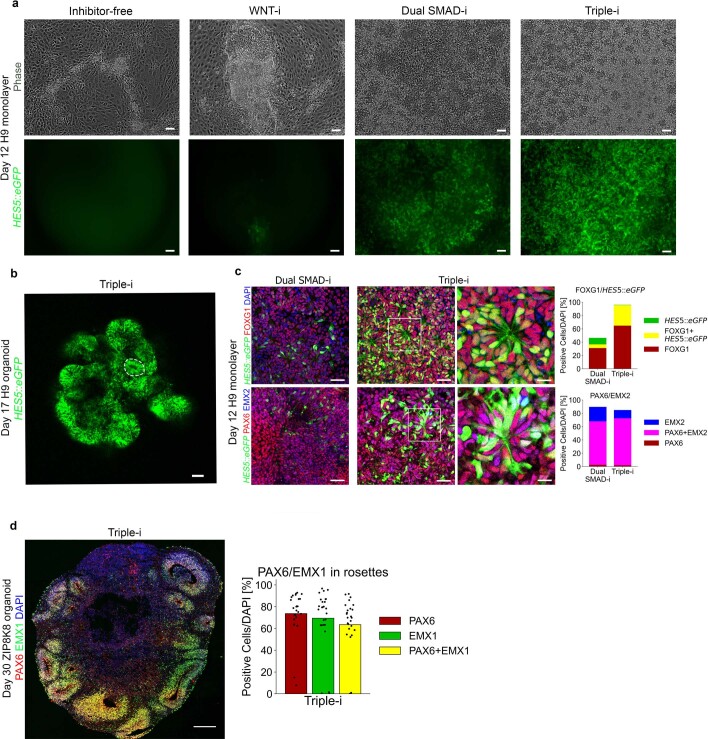
Enhanced Notch activation and efficient radial organization co-localize with cortical markers in Triple-i derived monolayer cultures. **a**. Phase contrast (top) and *HES5::eGFP* (bottom) images of representative day 12 neural monolayer progenitors derived from small EBs (sEBs) subjected to neural induction under Inhibitor-free conditions **(Inhibitor-free)**, WNT inhibition using XAV-939 **(WNT-i)**, Dual SMAD inhibition using SB-431542 and NOG **(Dual SMAD-i)** and combined Dual SMAD and WNT inhibition **(Triple-i)** (representative images from N=3 biological replicates for each protocol). Scale bar: 50 µm. **b**. Merged bright field images and matched H9 *HES5::eGFP* confocal image taken from a representative day 17 organoid derived under Triple-i protocol (representative image from N=9 biological replicates). Shown is an individual image from the z-stack of the representative organoid shown in Fig. 2a. A radially organized Notch active region is outlined in the dashed line. Scale bar: 100 µm. **c**. Immunostaining of FOXG1 (top) and PAX6 and EMX2 (bottom) with respect to Notch activation (*HES5::eGFP*) and radial organization in representative day 12 monolayer sEB protocol neural progenitors derived by indicated treatments (representative images from N=3 biological replicates for each protocol). The right image represents a magnified rosette from Triple-i derived progenitors. Scale bar: 50 µm. Cell counts and co-localization analysis of markers are presented in the right panel. See Source Data Extended Data Fig. 2 for immunostaining counts. **d**. Immunostaining for cortical markers PAX6 and EMX1 in Triple-i derived organoids generated using ZIP8K8 hiPSC line (representative images from N=2 biological replicates). Bars represent mean. Cell counts were generated from 26 rosettes across 2 organoids. Scale bar: 200 µm. See Source Data Extended Data Fig. 2 for immunostaining counts. Source data

Our bulk RNA-Seq analyses suggest that organoids with cortical fates are highly variable between and within methods unless Triple-i is employed. We looked into the cyto-architectural dynamics of NSCs in growing organoids in search of differential readouts across methods. By employing the *HES5::eGFP* NOTCH activation hESC line that reports for NSC activity^7,38^, it was highly evident that NSCs marked by NOTCH activation exhibit superior radial organization (rosette formation) capacity under Triple-i in 2D monolayer cultures (Extended Data Fig. 2a).

**Fig. 2:**
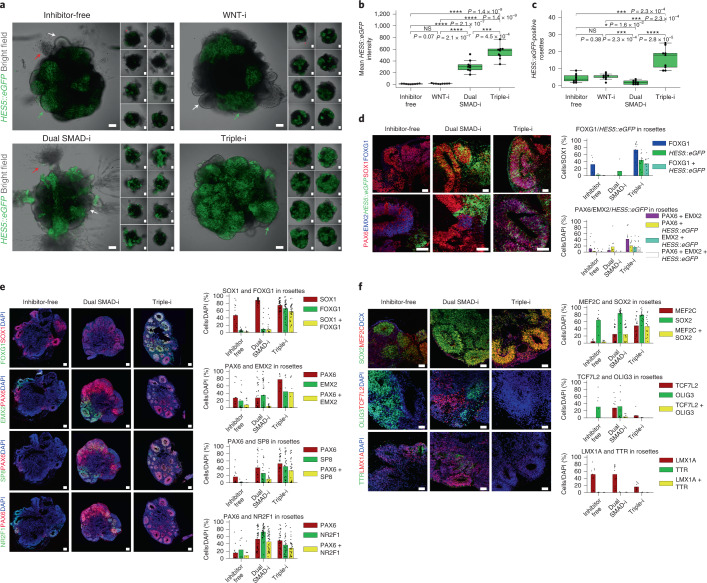
Enhanced NOTCH activation and efficient radial organization co-localize with cortical markers in organoids derived by Triple-i. **a**, Merged bright-field images and their matched H9 *HES5::eGFP* confocal images taken of representative day 17 organoids (*n* = 9). Maximum intensity projections for H9 *HES5::eGFP* confocal images taken at intervals of 15 µm are shown. Green arrows, enhanced NOTCH-active and radially organized regions; white arrows, radially organized regions lacking enhanced NOTCH activation; red arrows, non-neuro-epithelial extensions. **b**, Mean *HES5::eGFP* intensity levels measured for the images from **a** (*n* = 9). **c**, Number of rosettes expressing *HES5::eGFP* in the organoid images from **a**. **b**,**c**, Statistical test, two-sided *t*-test with Benjamini–Hochberg correction; *P < 0.05; ***P < 0.001; ****P < 0.0001; and NS, not significant. The boxplots display the median and interquartile range (box boundaries) with whiskers extending to 1.5× the interquartile range; *n* = 9. **d**, Immunostaining of representative H9-derived day 30 organoids (left) and counts within rosettes of SOX1, FOXG1 and *HES5::eGFP* (top right; Triple-i, *n* = 13 from two replicates; Dual SMAD-i, *n* = 4 from one replicate; and Inhibitor-free, *n* = 5 from one replicate) as well as PAX6, EMX2 and *HES5::eGFP* (bottom right; Triple-i and Dual SMAD-i, *n* = 10 from one replicate; and Inhibitor-free, *n* = 18 from two replicates). **e**, Immunostaining of representative ZIP8K8-derived day 27 organoids (left) as well as counts within rosettes of EMX2 (Triple-i, *n* = 8 from one replicate; Dual SMAD-i, *n* = 38 from three replicates; and Inhibitor-free, *n* = 14 from one replicate), SP8 (Triple-i, *n* = 35 from three replicates; Dual SMAD-i, *n* = 23 from three replicates; and Inhibitor-free, *n* = 12 rosettes from one replicate) and NR2F1 (Triple-i, *n* = 36 from three replicates; Dual SMAD-i, *n* = 44 from three replicates; and Inhibitor-free, *n* = 12 from one replicate) with PAX6, FOXG1 and SOX1 (Triple-i, *n* = 40 from two replicates; Dual SMAD-i, *n* = 29 from two replicates; and Inhibitor-free, *n* = 14 from one replicate; right). **f**, Immunostaining of representative ZIP8K8-derived day 27 organoids (left) and counts within rosettes of the cortical marker MEF2C with SOX2 and DCX (top right; Triple-i, *n* = 19 from two replicates; Dual SMAD-i, *n* = 22 from two replicates; and Inhibitor-free, *n* = 9 from one replicate), OLIG3 and TCF7L2 (middle right; Triple-i and Dual SMAD-i, *n* = 7 from one replicate; and Inhibitor-free, *n* = 11 from one replicate), and TTR and LMX1A (bottom right; Triple-i, *n* = 10; Dual SMAD-i, *n* = 13; and Inhibitor-free, *n* = 9; all from one replicate). **a**,**d**–**f**, Scale bars, 100 µm. **d**–**f**, The bars represent the mean. Immunostaining counts for **b–f** are provided. Source data

In organoids, radially organized regions reminiscent of VZ-like structures were observed under all treatments (Fig. 2a), in line with other studies^17,20,21,24–27,32,33^. However, collective NOTCH activation signals throughout the entire organoid volume in multiple organoids revealed that organoids derived under Inhibitor-free and WNT-i conditions displayed low NOTCH activation signals (Fig. 2b) and low numbers of NOTCH-active rosettes were observed (Fig. 2c). On the other hand, although both Dual SMAD-i and Triple-i organoids yielded regions with enhanced NOTCH activation (Fig. 2a,b), NOTCH activation was more restricted to radially organized structures in the Triple-i organoids (Fig. 2a,c and Extended Data Fig. 2b). These results demonstrate that early pathway inhibition had a robust effect on shaping radial organization—an important cyto-architectural feature of cortical NSCs.

To verify that NSCs in radially organized regions are of cortical identity, we performed a series of immunostainings of FOXG1, PAX6 and EMX2 in *HES5::eGFP* hESC organoids. FOXG1 was only partially expressed under Inhibitor-free conditions and it was completely absent in Dual SMAD-i organoids within rosettes, reflecting the non-cortical bias induced by these methods, whereas Triple-i organoids exhibited widespread FOXG1 expression in radially organized regions (vesicle areas) together with NOTCH activation (Fig. 2d). PAX6 and EMX2 are expressed in the cortex rostrally and caudally with shared regions dorsally^39^ but they are also expressed in non-cortical regions in the forebrain^40^. Accordingly, PAX6 and EMX2 expression was observed under all treatments (Fig. 2d), regardless of FOXG1 expression, implying both cortical and non-cortical identity. Only in Triple-i organoids and 2D monolayer cultures (Extended Data Fig. 2c) did both markers overlap to a large extent and coincide with the radial organization of NOTCH-active cells, linking rosette formation and dorsal cortical NSC identity.

The formation of these telencephalic VZ-like regions under Triple-i in hESC-derived organoids was further shown in human iPSCs. Rosette cells abundantly expressed FOXG1 in Triple-i iPSC organoids, whereas organoids generated through the other methods had few rosettes and lacked FOXG1 expression (Fig. 2e). The dorsal cortical marker EMX1 was observed throughout organoid vesicles in Triple-i organoids and co-localized with PAX6, together demarcating cortical VZ regions (Extended Data Fig. 2d). PAX6 and EMX2 as well as PAX6 and SP8, a rostrocaudal cortical marker, were widely co-expressed within rosettes only in Triple-i organoids (Fig. 2e). On the other hand, NR2F1, a distally located caudal cortical marker, was only moderately expressed in Triple-i organoids (Fig. 2e). Together with the previous findings in hESC organoids, these results suggest that Triple-i organoids display a pan-dorsal/medial cortical identity with a rostral bias. Furthermore, they underscore the necessity of confirming the co-expression of these markers in association with radial organization to verify cortical identity.

Finally, using immunostaining we validated some of the region-specific genes enriched in each derivation method based on the bulk RNA-Seq analysis. Whereas the Triple-i-enriched cortical marker MEF2C was detected in both radially organized and neuronal regions, non-cortical markers enriched in methods other than Triple-i—that is, OLIG3, TCF7L2, LMX1A and TTR—were found to be more widely expressed in organoids generated by the Inhibitor-free and Dual SMAD-i methods (Fig. 2f).

### Derivation methods evoke distinct NSC regional signatures

**Fig. 3:**
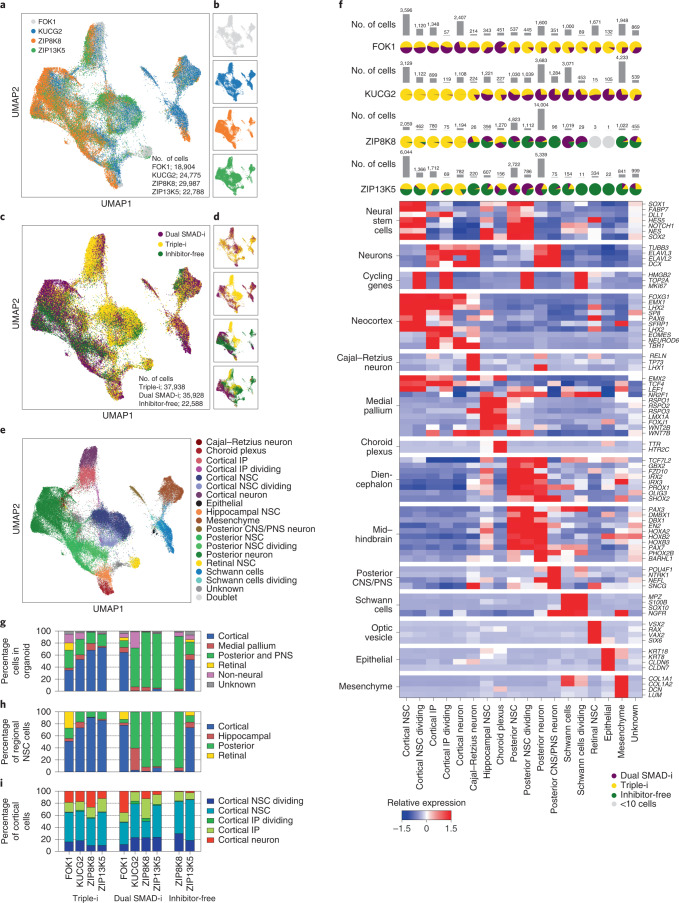
Triple-i treatment induces robust cortical identity and suppresses non-cortical fates across four iPSC lines. **a**, Uniform manifold approximation and projection (UMAP) generated from scRNA-Seq data of day 50 Triple-i organoids derived from FOK1, KUCG2, ZIP8K8 and ZIP13K5 iPSC lines; Dual SMAD-i organoids derived from FOK1, KUCG2, ZIP8K8 and ZIP13K5 iPSC lines; and Inhibitor-free organoids derived from ZIP8K8 and ZIP13K5 iPSC lines (*n* = 4 (FOK1 and KUCG2) and 5 (ZIP8K8 and ZIP13K5) organoids, pooled, from one replicate). The cells are coloured according to the respective iPSC line across all treatments. **b**, Cells from the individual iPSC lines were then plotted separately using the same UMAP embedding used in **a**. **c**, All day 50 organoid cells were plotted in the same UMAP embedding used in **a** and coloured according to the derivation protocol. **d**, Cells derived from Triple-i, Dual SMAD-i and Inhibitor-free derivation protocols were plotted using the same UMAP embedding used in **a**, separated according to the iPSC line as in **b**. **e**, Regional and cell-type annotations were then assigned to individual clusters (see Methods) and plotted on the same UMAP embedding. **f**, Heatmap of the relative expression values after *z*-score normalization of the average log-normalized expression values for each gene across annotated cell types after doublet removal for selected gene categories across all cells from **a**–**d** (bottom). The percentage of cells from each derivation protocol from all four iPSC lines are provided (top; pie charts). The pie charts are coloured in grey if fewer than ten cells from that iPSC line were assigned to the given cell type. The bar plots (top) display the total number of cells in each iPSC line assigned to the given cell type. **g**, Regional composition of all cells separated by cell line and derivation protocol. **h**, Regional composition of all NSCs separated by cell line and derivation protocol. **i**, Composition of all cortical cells separated by cell line and derivation protocol.

To further assess regional specification within NSCs, we employed scRNA-Seq on organoids from four iPSC lines. We analysed day 50 organoids to allow for the accumulation of diverse differentiated cell types (compared with day 30) and in particular to investigate whether cortical NSC identity was preserved long after inhibitor withdrawal. The single-cell transcriptomes exhibited a considerable overlap across all four iPSC lines (Fig. 3a,b), indicating the capability of all lines to give rise to similar cell types. However, whereas Triple-i cells distributed similarly irrespective of cell line, Dual SMAD-i and Inhibitor-free cells differentially partitioned depending on cell line, indicating that Triple-i enforced a robust effect on endogenous signalling (Fig. 3c,d).

**Extended Data Fig. 3:**
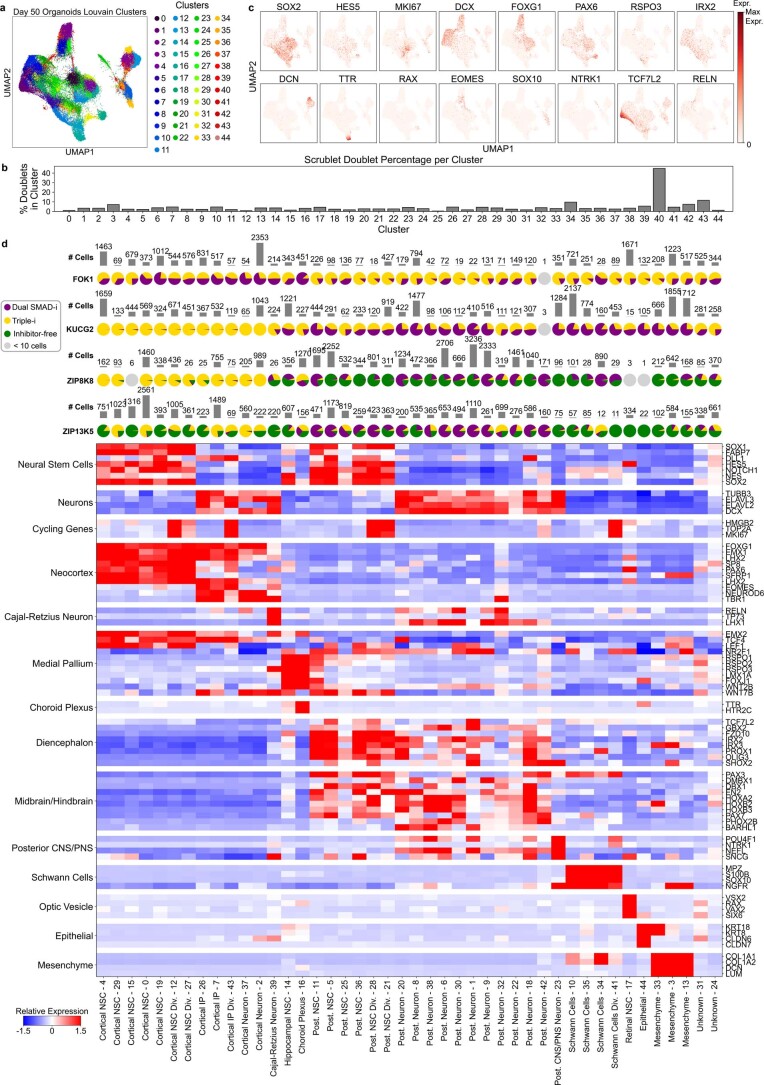
Clustering of scRNA-Seq data obtained from day 50 organoids across four cell lines and three protocols. **a**. A UMAP derived from scRNA-Seq data of day 50 Triple-i organoids derived from FOK1 (N=1 experiment, 4 organoids pooled), KUCG2 (N=1 experiment, 4 organoids pooled), ZIP8K8 (N=1 experiment, 5 organoids pooled) and ZIP13K5 (N=1 experiment, 5 organoids pooled) iPSC lines, Dual SMAD-i organoids derived from FOK1 (N=1 experiment, 4 organoids pooled), KUCG2 (N=1 experiment, 4 organoids pooled), ZIP8K8 (N=1 experiment, 5 organoids pooled) and ZIP13K5 (N=1 experiment, 5 organoids pooled) iPSC lines, and Inhibitor-free organoids derived from ZIP8K8 (N=1 experiment, 5 organoids pooled) and ZIP13K5 (N=1 experiment, 5 organoids pooled) iPSC lines. Each cell is coloured by its corresponding cluster estimated using Louvain clustering approach (see Methods). **b**. The relative percent of estimated doublets from Scrublet^67^ is plotted for each cluster. **c**. Expression levels in each cell for selected marker genes is plotted using the same UMAP embedding from (**a**). **d**. Heatmap displays relative expression values after z-score normalization of average log-normalized expression values for each gene across clusters after doublet removal (see Methods) for selected genes categorized according to neural stem, neuronal, cycling, regional, and other cell type markers. Pie charts above each cell type display percentage of cells from each derivation protocol across all 4 iPSC lines. Pie charts are coloured with a grey colour if less than 10 cells from that iPSC line were assigned to the given cluster. Bar plots display the total number of cells within each iPSC line assigned to the given cluster.

We then performed unsupervised clustering of all single-cell transcriptomes and identified a total of 45 clusters (Extended Data Fig. 3), which were then assigned to 18 cell types based on enrichments of known marker-gene expression (see Methods; Fig. 3e,f). We found that the organoids generated by Triple-i exhibited consistent and robust cortical specification across all four cell lines (median, 60%) accompanied by a repression of posterior and PNS fates (median, 23%). In stark contrast, three of the four cell lines differentiated under Dual SMAD-i exhibited an overwhelming posterior central nervous system (CNS)/peripheral nervous system (PNS) identity (median, 78%), whereas only one cell line (FOK1) contained a high level of cortical specification (64%). Inhibitor-free conditions also inconsistently gave rise to cortical populations, with one cell line (ZIP13K5) containing 52% cortical identity and the other (ZIP8K8) yielding merely 0.7% cortical identity (Fig. 3g).

Intriguingly, the preferential enrichment of cortical identity under Triple-i as well as the regional heterogeneity seen in Dual SMAD-i and Inhibitor-free conditions was strongly reflected in organoid NSC populations (Fig. 3h). These results demonstrate that early specification by exogenous cues evoked a regional bias that persisted in NSCs and their progeny as long as 40 days following inhibitor withdrawal. One of the most compelling pieces of evidence for the superiority of Triple-i is the ability of this method to produce nearly identical proportions of cortical germinal zone and neuronal cell populations across all cell lines (Fig. 3i).

**Extended Data Fig. 4:**
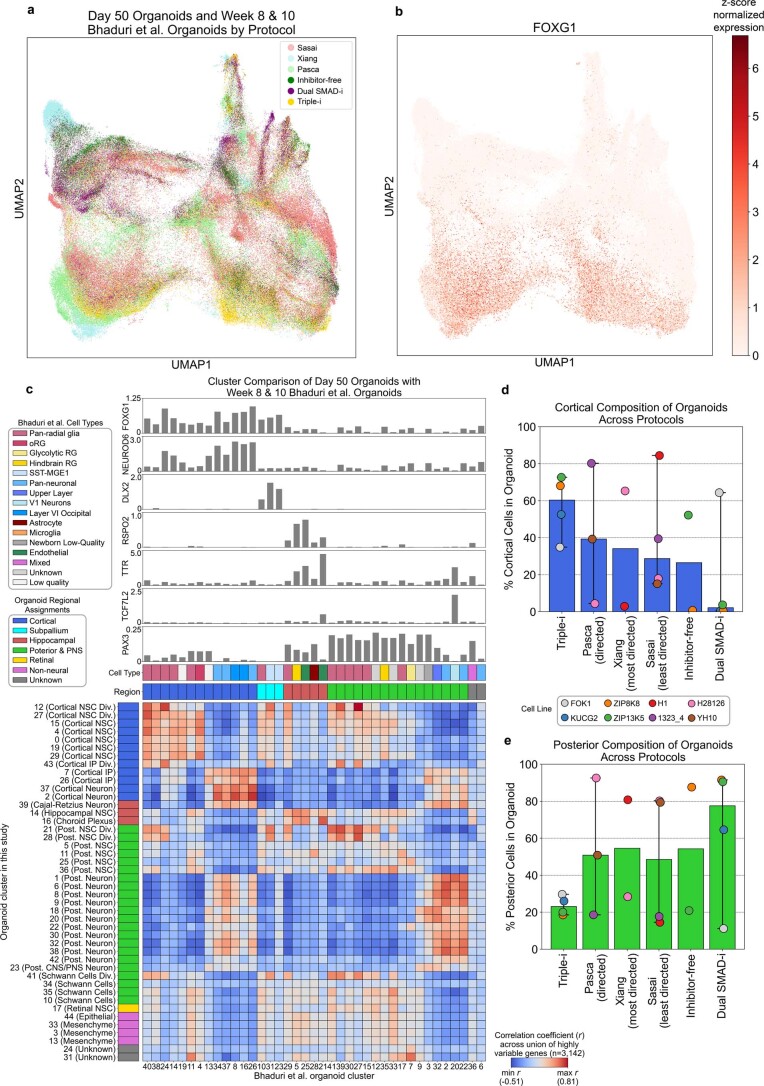
Integrated analysis with public datasets highlights consistent production of cortical cell types and repression of posterior cell types in Triple-i organoids. **a**. A UMAP derived from scRNA-Seq data of day 50 Triple-i, Dual SMAD-i and Inhibitor-free organoids from this study after doublet removal (see Extended Data Fig. 3a for description of all scRNA-Seq samples) after integrating with all week 8 and 10 in-vitro cells across 3 derivation methods from Bhaduri et al^31^.. Each cell is coloured by its respective derivation method. **b**. The z-score normalized expression levels of *FOXG1* across all cells was plotted using the same UMAP embedding from (**a**). **c**. Pearson correlation coefficients measured using average normalized expression levels of highly variable genes across all cells within a cluster between clusters found in this study and organoid clusters from weeks 8 and 10 found in Bhaduri et al. The bar plots above each column display the average expression levels across all cells within each Bhaduri et al. cluster of regional specific marker genes *FOXG1* (cortical/subpallium), *NEUROD6* (cortical neuron), *DLX2* (subpallium), *RSPO2* (hippocampal), *TTR* (choroid plexus), *TCF7L2* (diencephalon), *PAX3* (midbrain/hindbrain). **d**. The bar plot shows the median percentage of cells annotated as cortical across cell lines within each derivation protocol in both our scRNA-Seq dataset (Triple-i: N=4 experiments across 4 cell lines, Dual SMAD-i: N=4 experiments across 4 cell lines, Inhibitor-free: N=2 experiments across 2 cell lines) and Bhaduri et al. datasets (Pasca: N=3 experiments across 3 cell lines, Xiang: N=2 experiments across 2 cell lines, Sasai: N=4 experiments across 4 cell lines). Each dot shows the percentage of cortical cells within each cell line, and the intervals display the full range (minimum to maximum) of cortical percentages. Whiskers extend to minimum and maximum values when at least 3 samples present. **e**. The bar plot is the same as in (**d**) except shows the percentage of cells annotated as posterior/PNS across both datasets. Whiskers extend to minimum and maximum values when at least 3 samples present.

We next compared regional specification in organoids from this study with those obtained in a study conducted by Bhaduri and colleagues^31^ comparing stage-matched organoids generated side by side by various protocols^22,24,29^ and analysed them at the single-cell level. Merging of the datasets revealed a strong overlap of our Triple-i organoids with FOXG1^+^ populations across both studies (Extended Data Fig. 4a,b).

We then annotated clusters with brain regions from the Bhaduri study and observed a strong correlation between corresponding cell types across studies (Extended Data Fig. 4c). Importantly, based on these annotations we revealed that across all protocols and lines in both studies, our Triple-i organoids exhibited both the highest and most consistent levels of cortical-fate induction, accompanied by the strongest repression of posterior CNS/PNS fates (Extended Data Fig. 4d,e), further emphasizing the superiority of the Triple-i method in generating robust cortical organoids.

### Triple-i enriches for oRG cells demarcating oSVZ regions

**Fig. 4:**
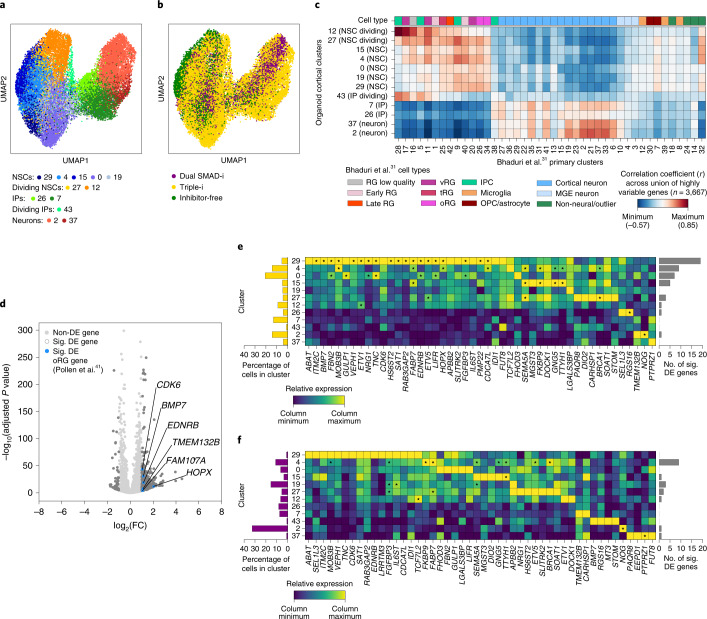
Triple-i organoids specifically enrich for oRG cells across four iPSC lines. **a**, UMAP of cortical cells derived from scRNA-Seq data of day 50 Triple-i, Dual SMAD-i and Inhibitor-free organoids derived from FOK1, KUCG2, ZIP8K8 and ZIP13K5 cell lines. See the Fig. 3a caption for a detailed description of the samples. Each cell is coloured by its respective cluster and annotated according to cell type. **b**, Cells derived from the Triple-i, Dual SMAD-i and Inhibitor-free protocols were plotted using the same UMAP embedding in **a**. **c**, Heatmap of the Pearson’s correlation coefficients (measured using average normalized expression levels of highly variable genes across all cells within a cluster) between the cortical clusters found in this study and the in vivo clusters found in the Bhaduri et al.^31^ study. RG, radial glia; vRG, ventral radial glia; tRG, truncated radial glia; oRG, outer radial glia; IPC, intermediate progenitor cells; OPC, oligodendrocyte progenitor cells; MGE, medial ganglionic eminence. **d**, Results of a differential gene expression analysis comparing all cortical NSCs from the Triple-i and Dual SMAD-i organoids from **a**. The log_2_-transformed fold-changes and adjusted *P* values from a *t*-test with overestimation of variance after Benjamini–Hochberg correction are shown (see Methods). Significantly differentially expressed oRG-specific marker genes derived from the Pollen et al.^41^ study are annotated and highlighted in blue. *P* = 0.0594 for gene-set enrichment analysis using a two-sided Fisher’s exact test of oRG genes within upregulated genes in Triple-i cortical NSCs. **e**, Heatmap showing the relative expression levels of all oRG-specific markers across cortical clusters in Triple-i organoids (middle). Significantly upregulated genes within a cluster are indicated with an asterisk. The percentage of cortical cells from Triple-i organoids in each cluster (left) and number of significantly upregulated genes per cluster (right) are shown. **f**, Heatmap and bar graph as in **d** after subsetting to cortical cells from Dual SMAD-i organoids. Sig. DE, significantly differentially expressed.

We next investigated whether oRG cells were preferentially enriched in Triple-i organoids. We first sought to identify clusters that exhibited strong similarities with in vivo oRG populations. A comparison of our cortical clusters (Fig. 4a,b) with the in vivo populations derived from the scRNA-Seq data of Bhaduri et al.^31^ highlighted several potential oRG populations, including clusters 29, 4 and 27 (Fig. 4c). A global differential expression analysis of all cortical NSC populations derived from all methods showed that oRG-specific marker genes extracted from a study conducted by Pollen and colleagues^41^ were enriched in the Triple-i-derived cortical NSCs when compared with those derived from Dual SMAD-i (Fig. 4d). Strikingly, among all Triple-i cortical cells, we found the strongest enrichment of oRG-specific marker genes in the cortical NSC cluster 29 (Fig. 4e). This enrichment was not present in Dual SMAD-i cells (Fig. 4f). Together, these findings indicate the ability of the Triple-i protocol to specifically enable the emergence of oRG cells.

**Fig. 5:**
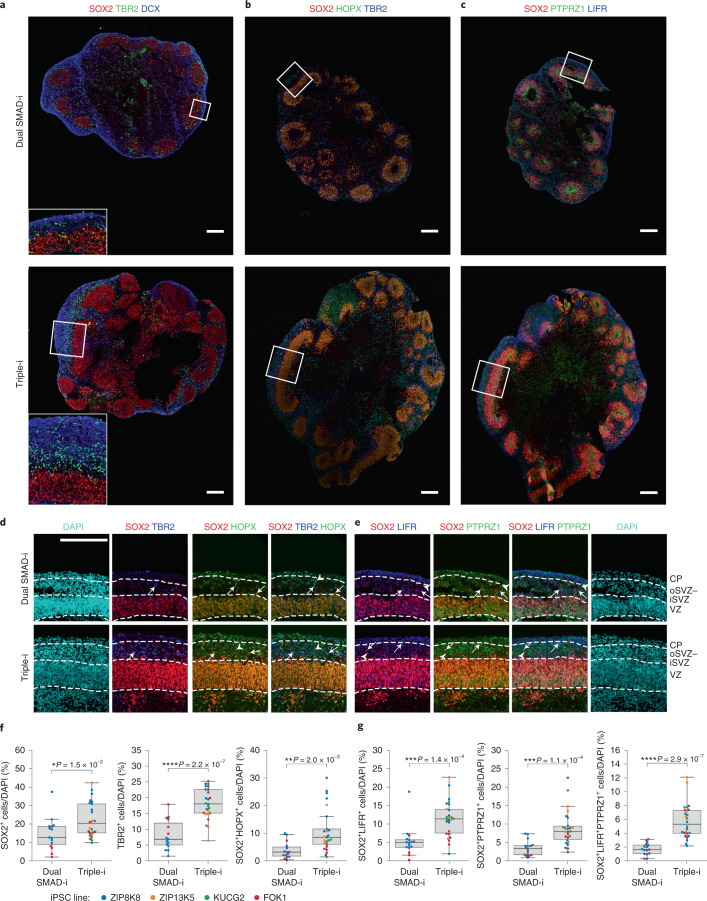
oRG cells demarcate oSVZ regions in Triple-i organoids. **a**, Immunostaining for SOX2, TBR2 and DCX in day 50 ZIP8K8 iPSC organoids derived under the indicated treatments. Inset: magnified view of the region in the white box in the main image (full organoid vesicle) in the respective treatment. **b**, Immunostaining for the iSVZ–oSVZ markers TBR2 and HOPX along with SOX2 in day 50 ZIP8K8 iPSC organoids derived under the indicated treatments. **c**, Immunostaining for the iSVZ–oSVZ markers LIFR and PTPRZ1 along with SOX2 in day 50 ZIP8K8 iPSC organoids derived under the indicated treatments. **d**, Magnified views of the region in the white boxes in **b** showing immunostaining for TBR2 and HOPX with respect to SOX2 under the indicated treatments. **e**, Magnified views of the region in the white boxes in **c** immunostained for LIFR and PTPRZ1 with respect to SOX2 under the indicated treatments. CP, cortical plate. **d**,**e**, The arrows and arrowheads represent tracking of specific cells with co-expression of different markers. **a**–**e**, Images are representative of immunostainings from two biological replicates for Triple-i (bottom) and Dual SMAD-i (top) organoids. Scale bars, 200 µm. **f**, Immunostaining cell counts and co-localization analysis for the iSVZ–oSVZ regions of Triple-i (*n* = 25 vesicles from eight replicates; two organoids per cell line) and Dual SMAD-i (*n* = 15 vesicles from four replicates; two organoids each for the FOK1 and ZIP8K8 cell lines, no vesicles were present in the ZIP13K5 and KUCG2 cell lines) organoids. **g**, Immunostaining cell counts and co-localization analysis for the iSVZ–oSVZ regions in Triple-i (*n* = 26 vesicles from eight replicates; two organoids per cell line) and Dual SMAD-i (*n* = 16 vesicles from four replicates; two organoids each for the FOK1 and ZIP8K8 cell lines, no vesicles were present in the ZIP13K5 and KUCG2 cell lines) organoids. **f**,**g**, Boxes display the median and interquartile range (box boundaries) with whiskers extending to 1.5× the interquartile range. Statistical test, two-sided *t*-test; *P < 0.05; **P < 0.01; ***P < 0.001; ****P < 0.0001. The immunostaining counts for **f**,**g** are provided. Source data

**Extended Data Fig. 5:**
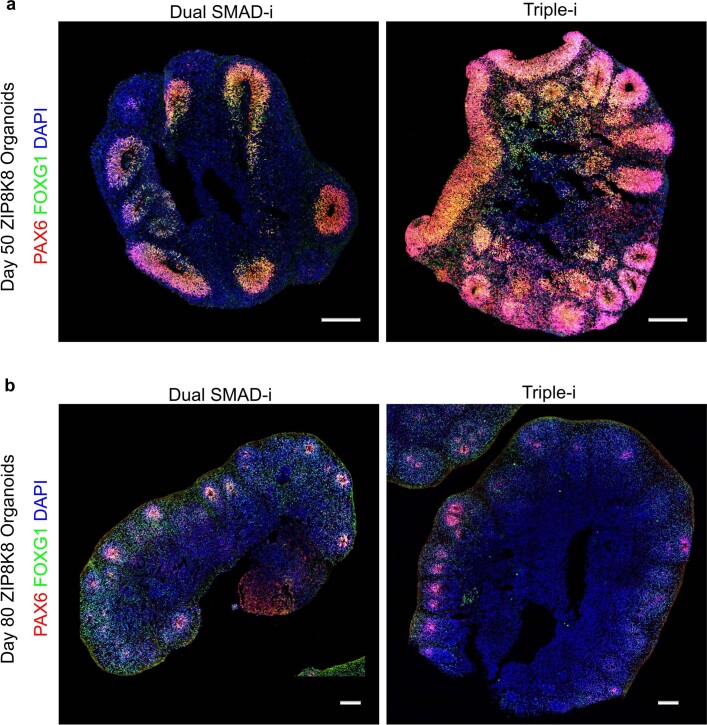
FOXG1 expression in mid-stage and late-stage ZIP8K8-derived Dual SMAD-i and Triple-i organoids. **a**. Immunostainings of cortical markers FOXG1 and PAX6 along with DAPI in representative day 50 ZIP8K8 iPSC organoids (N=2 Triple-i; N=2 Dual SMAD-i). Scale bar:200 µm. **b**. Immunostainings of cortical markers FOXG1 and PAX6 along with DAPI in representative day 80 ZIP8K8 iPSC organoids (N=2 Triple-i; N=2 Dual SMAD-i). Scale bar:200 µm.

**Extended Data Fig. 6:**
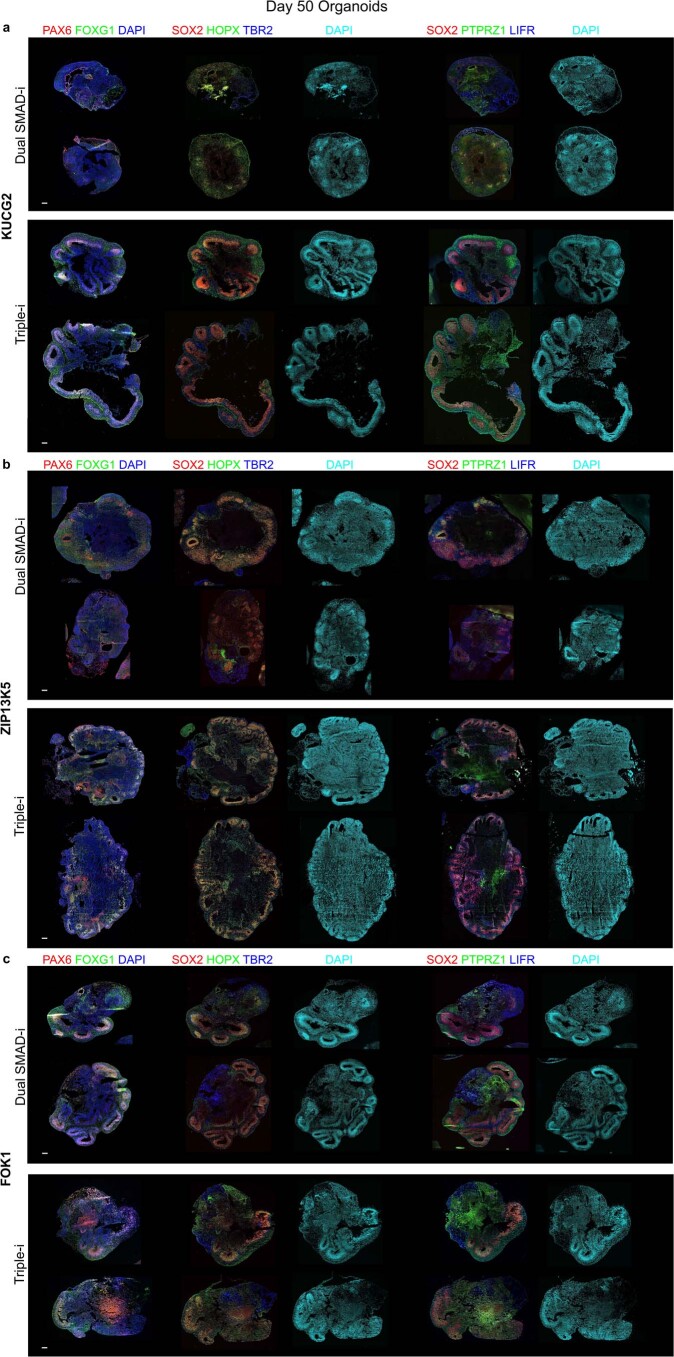
Immunostainings of day 50 organoids reveal robust generation of cortical cell types across multiple iPSC lines under Triple-i protocol. **a**. Immunostaining for cortical markers PAX6 and FOXG1, iSVZ/oSVZ markers TBR2 and HOPX along with SOX2, and oRG markers LIFR and PTPRZ1 along with SOX2 in day 50 Dual SMAD-i organoids (N=2) and day 50 Triple-i organoids (N=2) derived from the KUCG2 iPSC line. Scale bar: 200 µm. Note the lack of FOXG1 expression in both Dual SMAD-i organoids and prominent FOXG1+ rosettes in Triple-i organoids. **b**. Immunostaining for cortical markers PAX6 and FOXG1, iSVZ/oSVZ markers TBR2 and HOPX along with SOX2, and oRG markers LIFR and PTPRZ1 along with SOX2 in day 50 Dual SMAD-i organoids (N=2) and day 50 Triple-i organoids (N=2) derived from the ZIP13K5 iPSC line. Scale bar: 200 µm. Note the lack of FOXG1 expression in both Dual SMAD-i organoids and widespread FOXG1+ rosettes in Triple-i organoids. **c**. Immunostaining for cortical markers PAX6 and FOXG1, iSVZ/oSVZ markers TBR2 and HOPX along with SOX2, and oRG markers LIFR and PTPRZ1 along with SOX2 in day 50 Dual SMAD-i organoids (N=2) and day 50 Triple-i organoids (N=2) derived from the FOK1 iPSC line. Scale bar: 200 µm. Note the presence of FOXG1 expression in both Dual SMAD-i and Triple-i organoids co-localizing with rosette structures.

Finally, we utilized some of the established Pollen^41^ oRG markers to assess the spatial expression pattern of oRG cells surrounding radially organized VZ regions in day 50 Dual SMAD-i and Triple-i organoids across the four cell lines analysed by scRNA-Seq. We first assessed the cortical identity of the organoids by FOXG1 staining. For the Triple-i-derived organoids, all four cell lines expressed FOXG1 within and surrounding radially organized PAX6 and SOX2-expressing VZ regions (Extended Data Figs. 5 and 6). In contrast, FOXG1 expression in Dual SMAD-i organoids could only be observed for two cell lines (FOK1 and ZIP8K8; Extended Data Figs. 5 and 6). These findings validate the consistent cortical NSC identity in Triple-i organoids across all cell lines, as shown by scRNA-Seq. We next examined regions outside the VZ and found that across all four lines, Triple-i organoids had a higher number of TBR2^+^ intermediate progenitor (IP) cells that also formed sizeable SVZ-like structures (Fig. 5a,b,f).

We evaluated the contribution of the derivation methods to oRG cells by looking at SOX2^+^ cells surrounding the VZ regions. We found that SOX2 and HOPX-expressing cells, potentially marking oRG NSCs, occurred at higher proportions in Triple-i organoids (Fig. 5b,d,f and Extended Data Fig. 6). Moreover, these oRG cells were contained within a visible region demarcated by PTPRZ1—possibly signifying an oSVZ region (Fig. 5c,e)—whereas this region was poorly defined in Dual SMAD-i organoids. Similarly, we found a higher proportion of SOX2^+^ cells co-localizing with either LIFR or PTPRZ1, or LIFR and PTPRZ1 combined in these iSVZ–oSVZ regions in the Triple-i organoids compared with the Dual SMAD-i organoids (Fig. 5g). Notably, HOPX, PTPRZ1 and LIFR were also widely expressed in the VZ regions, further underscoring the necessity to include both spatial and molecular information to identify bona fide oRG cells based on these markers.

To conclude, these findings validate the enhanced oRG gene signature in Triple-i organoids detected in scRNA-Seq at the cyto-architectural level and further demonstrate the marked increase in the presence of oRG cells specifically within the iSVZ–oSVZ regions across four cell lines. Together, these findings highlight the ability of the Triple-i method to reproducibly generate an enriched NSC cortical identity that corresponds well with a marked diversification of germinal zone cells across different cell lines.

### Triple-i organoids reproduce cortical cellular diversity

**Fig. 6:**
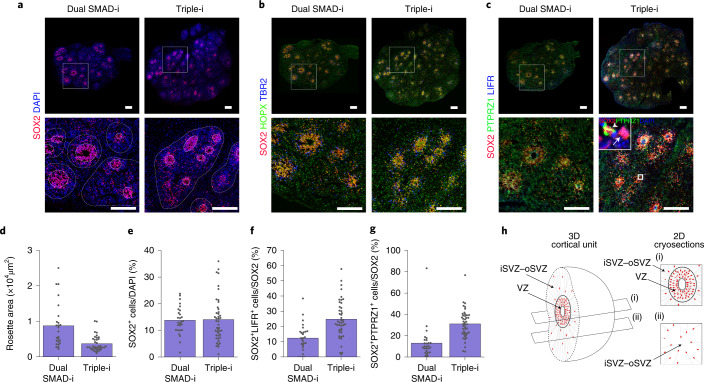
Later-stage Triple-i organoids exhibit homogenous cortical units and enriched oRG cell populations. **a**, Immunostaining for the stem cell marker SOX2 and 4,6-diamidino-2-phenylindole (DAPI) in day 80 ZIP8K8 iPSC organoids (top). Magnified views of the boxed region from the full organoid vesicle are shown (bottom). Cortical units are encircled by a dashed line and VZ regions by a solid line. **b**, Immunostaining for the iSVZ and oSVZ markers TBR2 and HOPX as well as SOX2 in day 80 ZIP8K8 iPSC organoids (top). Magnified views of the regions in the white boxes are shown (bottom). **c**, Immunostaining for the oRG markers LIFR and PTPRZ1 as well as SOX2 in day 80 ZIP8K8 iPSC organoids (top). Magnified views of the regions in the white boxes are shown (bottom). Inset (bottom right): ×10 zoom-in of the outlined region in the image for a Triple-i organoid (top right) highlighting SOX2^+^PTPRZ1^−^ (arrow) and SOX2^+^PTPRZ1^+^ (arrowhead) cells. **a**–**c**, Representative images of immunostainings of two biological replicates for Triple-i and Dual SMAD-i organoids, with similar results. Scale bars, 200 µm. **d**, Size of the VZ in Dual SMAD-i (*n* = 25 from two replicates) and Triple-i (*n* = 37 from two replicates) organoids derived from ZIP8K8 iPSCs. **e**, Proportion of SOX2^+^ cells per DAPI within cortical units inside the iSVZ–oSVZ regions in Dual SMAD-i and Triple-i organoids. **f**, Proportion of SOX2^+^LIFR^+^ cells per SOX2 in the same cortical units in **e**. **g**, Proportion of SOX2^+^PTPRZ1^+^ cells per SOX2 in the same cortical units as in **e**. **e**–**g**, Dual SMAD-i, *n* = 27 from two replicates; and Triple-i, *n*  = 44 from two replicates. **d**–**g**, The bars represent the mean. **h**, Schematic representing the VZ and iSVZ–oSVZ areas from a single cortical unit in cryosectioned organoids. Many cortical units contained purely interspersed SOX2^+^ cells alongside TBR2^+^ IP cells but lacked well-defined rosettes, suggesting that these units were cryosectioned through their oSVZ regions. Immunostaining counts for **d**–**g** are provided. Source data

**Fig. 7:**
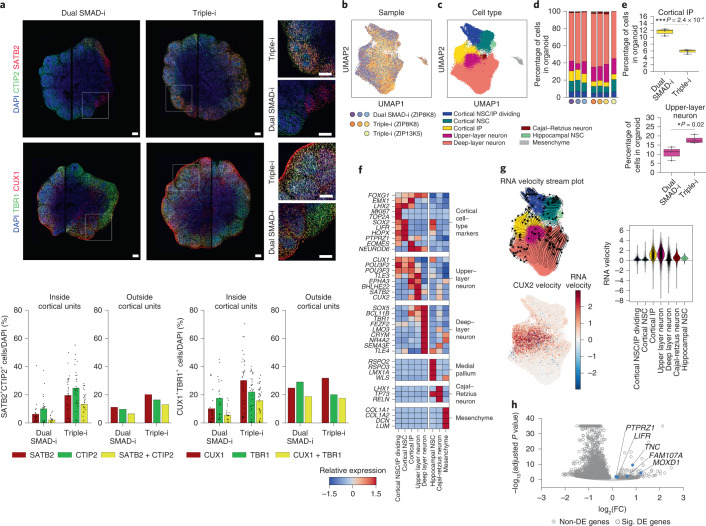
Later-stage Triple-i organoids exhibit molecularly distinct upper- and deep-layer neurons. **a**, Immunostaining for TBR1 and CUX1, and CTIP2 and SATB2 in day 80 ZIP8K8 organoids (top left). Magnified views of the boxed regions in the main images are shown (top right). Immunostaining counts for TBR1 and CUX1 (bottom; Dual SMAD-i, *n* = 21 cortical units; and Triple-i, *n* = 34 cortical units; one replicate for both groups), and CTIP2 and SATB2 (Dual SMAD-i, *n* = 21 cortical units; and Triple-i, *n* = 29 cortical units; one replicate for both groups). The bars represent the mean. Scale bars, 200 µm. **b**, UMAP derived from scRNA-Seq data of day 80 ZIP13K5 Triple-i pooled organoids (*n* =3 organoids pooled from one experiment), and ZIP8K8 individual Triple-i (*n* = 3) and Dual SMAD-i (*n* = 3) organoids. Cells are coloured according to the sample. **c**, Cell-type annotations using the same UMAP embedding in **b**. **d**, Cell-type compositions from each scRNA-Seq experiment. Experiments and cell-types are colour-coded as in **b**,**c**, respectively. **e**, Comparison of the proportion of cortical IP (top) and upper-layer neurons (bottom) in the scRNA-Seq experiments shown in **b** for Dual SMAD-i (*n* = 3 replicates) and Triple-i (*n* = 4 replicates) day 80 organoids. Boxes display the median and interquartile range (box boundaries) with whiskers extending to 1.5× the interquartile range. Statistical test, two-sided *t*-test; *P < 0.05 and ***P < 0.001. **f**, Heatmap displaying the relative expression values after *z*-score normalization of the average log-normalized expression values for each gene across cell types derived from all samples in **b**–**d**. **g**, RNA velocity stream plot for day 80 Dual SMAD-i and Triple-i organoids (top left) along with the RNA velocity estimates for *CUX2* (bottom left). *CUX2* RNA velocity estimates per cell type (right). **h**, Differential gene expression analysis comparing all cortical NSCs from all Triple-i ZIP8K8 and ZIP13K5 organoids described in **b** (*n* = 4 replicates in total) with all ZIP8K8 Dual SMAD-i organoids described in **b** (*n* = 3 replicates in total), with selected oRG genes highlighted in blue. The log_2_-transformed fold-change and adjusted *P* values from a *t*-test with overestimation of variance after Benjamini–Hochberg correction are shown (see Methods). *P* = 1.2 × 10^−10^ for gene-set enrichment analysis using a two-sided Fisher’s exact test of oRG genes in Triple-i cortical NSCs. Sig., significant; DE, differential gene expression. Immunostaining counts for **a** are provided. Source data

**Extended Data Fig. 7:**
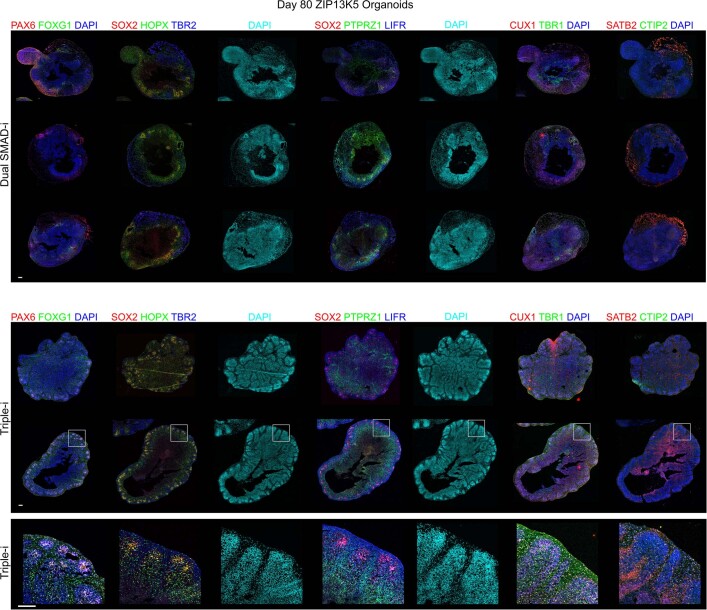
Immunostainings of later stage ZIP13K5 iPSC derived organoids highlight the pervasive presence of cortical units in Triple-i organoids. Immunostaining for cortical markers PAX6 and FOXG1, iSVZ/oSVZ markers TBR2 and HOPX along with SOX2, oRG markers LIFR and PTPRZ1 along with SOX2, upper layer neuronal marker CUX1 along with deep layer neuronal marker TBR1, and upper layer neuronal marker SATB2 along with deep layer neuronal marker CTIP2 in day 80 Dual SMAD-i organoids (N=3) and day 80 Triple-i organoids (N=2) derived from the ZIP13K5 iPSC line. Scale bar: 200 µm. Zoom-ins of the bottom Triple-i organoid are shown in the bottom panel. Note the lack of FOXG1 expression across all three Dual SMAD-i organoids and the presence of cortical units covering both Triple-i organoids, with co-localization of SOX2 and oRG markers, as well as interspersed upper and deep layer neuronal gene expression.

**Extended Data Fig. 8:**
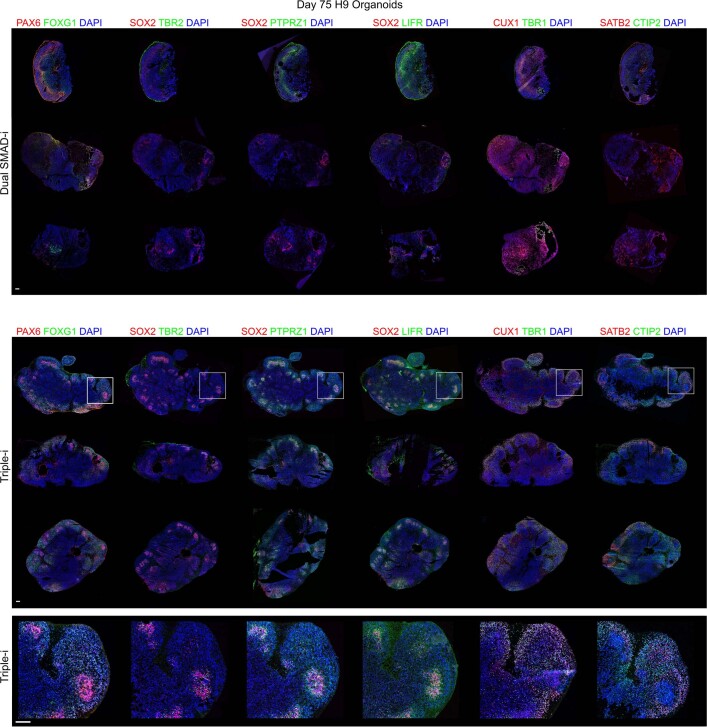
Immunostainings of later stage H9 hESC derived organoids highlight the pervasive presence of cortical units in Triple-i organoids. Immunostaining for cortical markers PAX6 and FOXG1, NSC marker SOX2 along with IP marker TBR2, NSC marker SOX2 along with oRG markers LIFR and PTPRZ1, upper layer neuronal marker CUX1 along with deep layer neuronal marker TBR1, and upper layer neuronal marker SATB2 along with deep layer neuronal marker CTIP2 in day 75 Dual SMAD-i organoids (N=3) and day 75 Triple-i organoids (N=3) derived from the H9 hESC line. Scale bar: 200 µm. Zoom-ins of the top Triple-i organoid are shown in the bottom panel. Note the lack of FOXG1 expression in the Dual SMAD-i organoid and the presence of cortical units covering the entire Triple-i organoid, with co-localization of SOX2 and oRG markers, as well as interspersed upper and deep layer neuronal gene expression.

We further investigated whether the oRG signature in Triple-i organoids persisted at a later stage of organoid development across three different cell lines. We first identified discrete regions of dense nuclei separated by areas consisting of low nuclei density (Fig. 6a; illustrated in Fig. 6h). These dense regions strictly co-localized with FOXG1 expression and consisted of NSCs expressing oRG markers (Fig. 6b,c and Extended Data Figs. 7,8). SOX2^+^ cells were present in these regions, both luminally within rosettes as well as basally interspersed along with TBR2^+^ IP cells and neurons, but were rarely detected beyond these dense areas. This was in contrast to neurons, which were also found beyond these regions (Extended Data Figs. 7 and 8; see also Fig. 7a). This suggested that these dense-nuclei regions, which we termed cortical units, represented distinctive in vitro counterparts to the VZ and potential iSVZ–oSVZ germinal zones, whereas areas beyond these regions mirrored more cortical plate-like regions. We found that Triple-i-derived organoids across all three cell lines (ZIP13K5, ZIP8K8 and H9) assessed on day 80 were predominantly comprised of these cortical units. In contrast, only ZIP8K8 organoids produced cortical units under Dual SMAD-i (Extended Data Fig. 5b), whereas ZIP13K5 and H9 Dual SMAD-i organoids completely lacked or showed sporadic FOXG1 expression (Extended Data Figs. 7 and 8). These results show that cortical identity at later stages is associated with higher cyto-architectural organization of multiple autonomic cerebral structures developing from early rosettes.

We next assessed the added impact of WNT-i on Dual SMAD-i in reproducing oRG cells in late-stage organoids, particularly in ZIP8K8 organoids—the only cell line that could form cortical units under Dual SMAD-i. SOX2^+^ rosettes in Dual SMAD-i cortical units were variable in size, whereas they were predominantly uniform in size and thin in Triple-i cortical units (Fig. 6d), suggesting more developmental synchroneity under Triple-i. Concordantly, whereas the iSVZ–oSVZ regions contained similar levels of SOX2^+^ cells across both treatments (Fig. 6e), Triple-i-derived cortical units contained higher levels of SOX2^+^ cells co-expressing PTPRZ1 and LIFR (Fig. 6f,g), suggesting an enhanced oRG specification. Together, these findings elucidate the ability of the Triple-i method to reproduce and maintain cortical identity and cortical NSC diversification across different cell lines at later stages of development.

The robust effect of Triple-i on the potency of NSCs within cortical units was further manifested by the widespread enrichment of upper- and deep-layer neurons. In ZIP8K8 organoids derived by Triple-i, the deep- and upper-layer neuronal markers CTIP2 and SATB2 were expressed at higher levels both separately and together, and more uniformly within and outside the cortical units, marking newly born migrating neurons in germinal zones as well as accumulated neurons in cortical plate-like regions (Fig. 7a). Co-expression of deep- and upper-layer markers has been observed in vivo in the maturing prefrontal cortex and thus may replicate in vivo development^42^. The expression patterns of these markers were also recapitulated in ZIP13K5 and H9 Triple-i organoids, whereas only sporadic expression of these markers was detected in ZIP13K5 and H9 Dual SMAD-i organoids, which also lacked FOXG1 expression (Extended Data Figs. 7 and 8). The upper-layer neuronal marker CUX1 was present in Triple-i organoids within and outside cortical units among all lines (Fig. 7a and Extended Data Figs. 7,8), in agreement with its expression within the VZ and SVZ regions in the developing brain before its expression in the upper neuronal layers^43^. Although this marker was also expressed across all Dual SMAD-i organoids, it was not associated with the presence of cortical units or FOXG1 expression under this treatment. Interestingly, ZIP8K8 cortical units showed minimal difference in the expression of TBR1 across the two methods, reflecting a comparable accumulation of this early neuronal marker. Conversely, ZIP13K5 and H9 Dual SMAD-i-derived organoids lacked TBR1 expression in comparison to their Triple-i counterparts (Extended Data Figs. 7 and 8), in correlation with the lack of cortical units in these Dual SMAD-i organoids. Together, these findings show that the Triple-i method is capable of generating a more enhanced cortical neuronal diversification in comparison to Dual SMAD-i.

To further investigate the enhanced cortical neuronal diversification and oRG specification in Triple-i organoids, we performed scRNA-Seq of day 80 organoids in ZIP8K8 and ZIP13K5 cell lines (Fig. 7b). As determined through immunostaining, these organoids contained predominantly cortical cell populations (Fig. 7c). Both the Dual SMAD-i and Triple-i organoids exhibited a remarkable reproducibility with respect to their cellular composition across protocols and cell lines (Fig. 7d). In addition, Triple-i-derived organoids contained a significantly higher number of upper-layer neurons and significantly lower number of IP cells than their Dual SMAD-i counterparts (Fig. 7e), confirming our findings derived from immunostaining and suggesting a less differentiated stage in Dual SMAD-i organoids. Deep and upper cortical neuronal-layer-specific markers^44,45^ were enriched in distinct subpopulations, with NEUROD6, a marker for newly born cortical neurons, being more highly expressed in the upper-layer neurons and IP cells, suggesting a bias towards upper-layer neurogenesis (Fig. 7f). This was further supported by the expression of upper-layer neuronal markers in NSCs and IPs (Fig. 7f). Moreover, the spliced form of CUX2 was most abundant in the upper-layer neurons, whereas the rate of *CUX2* gene transcription was similarly abundant in IP cells and upper-layer neurons, in line with its expression in the SVZ regions^43^ (Fig. 7g), suggesting a direct IP-to-upper-layer neuron transition. Finally, Triple-i cortical NSCs exhibited a pronounced upregulation of oRG-specific marker genes derived from Pollen et al.^41^ when compared with Dual SMAD-i cortical NSCs (two-sided Fisher’s exact test, *P* = 1.2 × 10^−10^; Fig. 7h). When combined with immunostaining, these results argue that Triple-i organoids at this later stage of development not only exhibit a more pronounced upper-layer neurogenesis but also continue to enrich for oRG cell populations.

### Triple-i microcephaly organoids model cortex-specific phenotypes

**Extended Data Fig. 9:**
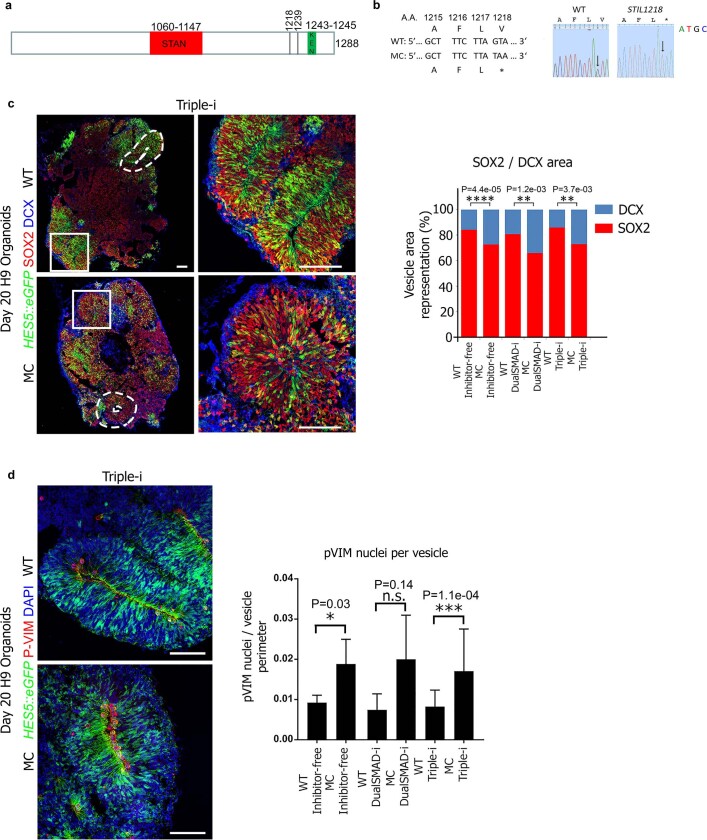
Microcephaly transcriptomic and cellular analyses. **a**. Description of the STIL protein including the STAN and KEN domains, as well as Microcephaly mutations. **b**. Normal and planned mutated nucleotide sequence adjacent to the region 183 corresponding to the 1218a.a (left). Genome edited sequencing results (right). The generated mutation is homozygous. **c**. Combined *HES5:eGFP* expression and immunostaining images for the neural progenitor marker SOX2 and the early neuronal marker DCX in representative day 20 WT and MC mutant organoids (representative images from N=3 biological replicates for WT and MC organoids) derived under Triple-i conditions. Four-fold magnifications of regions in left images are shown on the right. Quantification of SOX2 and DCX area proportions within vesicles obtained WT or MC organoids derived by indicated treatments and derived from N=3 organoids in each treatment. Scale bar:100 µm. Statistics: Stacked columns represent relative SOX2 (red) and DCX (blue) expression within selected areas from WT and mutant vesicles. Statistical test: *two-sided t-test*; *P<0.05;**P<0.01;***P<0.001;****P<0.0001. See Source Data Extended Data Fig. 9 for immunostaining counts. **d**. Combined *HES5:eGFP* expression and immunostaining images for the dividing RG marker P-VIM along with their corresponding DAPI images in representative day 20 WT and MC mutant organoids (representative images from N=3 biological replicates for WT and MC organoids) derived under Triple-i conditions. Right: cell counts of luminal P-VIM in WT and MC organoids derived by indicated treatments are derived from N=3 organoids in each treatment. Whiskers extend to minimum and maximum values. Statistical test: *two-sided t-test*; *P<0.05;**P<0.01;***P<0.001;****P<0.0001. See Source Data Extended Data Fig. 9 for immunostaining counts. Source data

Our findings predict that iPSC-based cortical-disease modelling systems relying on diverse methods are projected to yield distinct disease phenotypes. To provide a proof-of-concept for this idea, we generated a homozygous microcephaly mutation in the same isogenic *HES5::eGFP* reporter line used in this study. This mutation was generated by a guanine deletion at amino-acid position 1218 of the centriolar gene *STIL*, resulting in a truncated protein (Extended Data Fig. 9a,b) known to eventually cause autosomal recessive microcephaly in humans^46^.

**Fig. 8:**
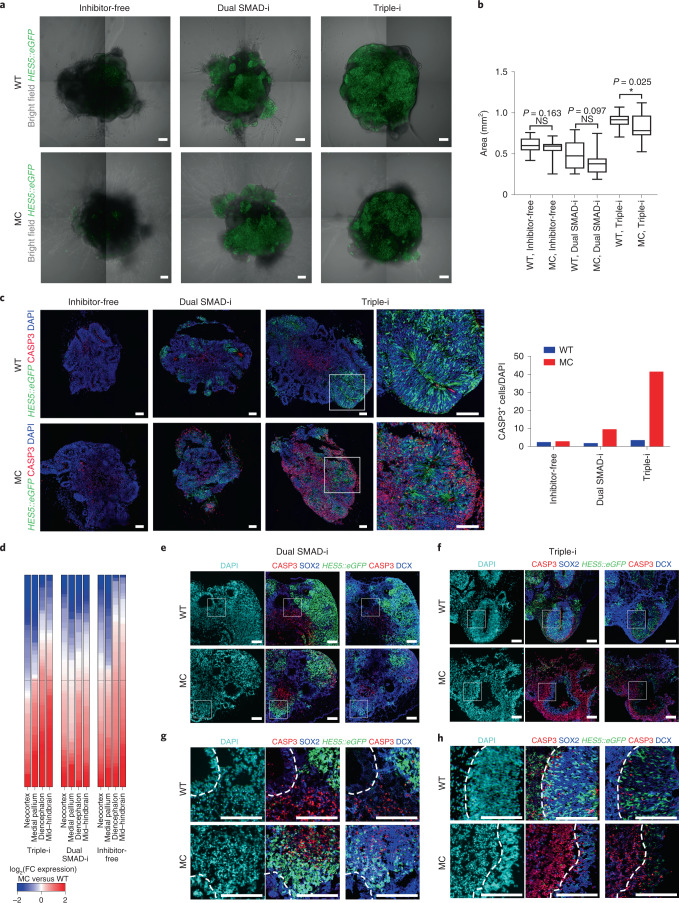
Differential phenotypic modelling of microcephaly organoids in the H9-derived *HES5:eGFP* hESC line by diverse pathway inhibition paradigms. **a**, Merged bright-field images and their matched *HES5:eGFP* confocal images obtained from representative day 17 organoids derived from WT and microcephaly (MC)-mutant H9 hESCs (Inhibitor-free, *n* = 25 (WT) and 15 (MC) organoids; Dual SMAD-i and Triple-i, *n* = 24 (WT) and 16 (MC) organoids). Maximum intensity projections of *HES5::eGFP* confocal images obtained at 6-µm intervals across each organoid are shown. **b**, Organoid sizes of day 17 WT and MC organoids, obtained by measuring the areas of the collapsed maximum intensity projection images from **a**. Boxplots display the median and interquartile range (box boundaries) with whiskers extending to 1.5× the interquartile range (see **a** for the number of replicates). Statistical test, two-sided *t*-test; *P < 0.05 and NS, not significant. **c**, Combined *HES5::eGFP* expression and immunostaining images of the apoptotic marker CASP3 in day 20 WT and MC organoids (*n* = 1 biological replicate per protocol). Magnified views (×3) of the regions in the white boxes (Triple-i-derived organoids) are shown (last column of images). Number of CASP3^+^ in WT and MC organoids (right). **d**, Heatmap showing log_2_-transformed fold changes in the expression levels of regional genes, derived from Allen Human Brain Atlas samples, measured between pooled homozygous MC and WT organoids (*n* = 1 biological replicate per protocol). Genes are ordered according to the log_2_-transformed fold-change value of each gene set, obtained from bulk RNA-Seq, and the line displays the midpoint. **e**, Combined *HES5::eGFP* expression and immunostaining images of the apoptotic marker CASP3 together with SOX2 (middle) and DCX (right) from an adjacent slice in Dual SMAD-i WT and MC day 30 organoids (*n* = 1 biological replicate). **f**, Combined *HES5::eGFP* expression and immunostaining images of the apoptotic marker CASP3 together with SOX2 (middle) and DCX (right) from an adjacent slice in Triple-i WT and MC day 30 organoids (*n* = 1 biological replicate). **e**,**f**, The DAPI immunostaining corresponds to SOX2. **g**,**h**, Magnified (×4) views of the regions in the white boxes in **e**,**f**, respectively. Dashed lines encircle VZ areas in WT and MC organoids. **a**,**c**,**e**–**h**, Scale bars, 100 µm (**a**,**c**) and 200 µm (**e**–**h**). The organoid sizes for **b** and immunostaining counts for **c** are provided. Source data

We found that day 17 microcephaly organoids were significantly smaller than wild-type (WT) organoids when derived using the Triple-i method (Fig. 8a,b). In agreement with this, Triple-i microcephaly organoids showed substantial expression of the apoptotic marker activated CASP3 mainly surrounding NOTCH-active radially organized regions; this marker was nearly absent in Inhibitor-free and only sparsely present in Dual SMAD-i microcephaly organoids (Fig. 8c). These results together suggest that the cell loss potentially causing the smaller organoid size under Triple-i was of cortical identity.

Integration of bulk RNA-Seq data of day 17 microcephaly organoids into the correspondence analysis plot in Fig. 1a shows that microcephaly samples cluster together regardless of method, similar to WT organoids, indicating that early disease phenotypes could not be well reflected at the global transcriptome level. On the other hand, day 30 microcephaly organoids clustered according to method (Fig. 1a), suggesting that regional specification dictated by protocols occurred in the presence of the STIL mutation. Interestingly, however, day 30 Triple-i microcephaly samples clustered most distally, adjacent to Dual SMAD-i organoids, implying that microcephaly organoids derived by Triple-i exhibit reduced cortical identity and elevated posterior-fate acquisition. This was further substantiated by a decrease in the expression of cortex-specific genes, accompanied by an increase in the expression of mid–hindbrain genes in Triple-i microcephaly organoids (Fig. 8d). Moreover, this phenotype only occurred under Triple-i conditions, demonstrating that Triple-i is the preferential method to reveal specific loss of cortical identity in STIL-mutated microcephaly organoids.

In contrast, some phenotypes in microcephaly organoids were detected irrespective of the method, such as a decline in ventricular versus neuronal area ratios judged by SOX2 and DCX, respectively (Extended Data Fig. 9c), as well as the accumulation of dividing cells within apical ventricular linings (Extended Data Fig. 9d), implying increased neurogenesis and cell-cycle defects.

We further characterized apoptotic cells with respect to the derivation method in day 30 microcephaly organoids. Immunostaining for CASP3 on day 30 revealed that similar to day 20 organoids, cell death was predominant in Triple-i organoids (Fig. 8f,h). The Triple-i organoids showed cell death (Fig. 8h) mainly at vesicle peripheries, basally to SOX2^+^ NOTCH-active cells, with compromised radial organization, suggesting that cell death encompassed SVZ and neuronal areas. In contrast, cell death in Dual SMAD-i organoids occurred irrespective of NOTCH activation or radial organization (Fig. 8e,g). Immunostaining with DCX showed that basally located CASP3^+^ cells in Triple-i organoids overlapped with neuronal cells (Fig. 8h). Interestingly, these CASP3^+^ areas in the Triple-i organoids strictly overlapped with a punctate DAPI pattern, indicating nuclear fragmentation, suggesting late-stage apoptosis of neuronal cells (Fig. 8h). Thus, together with our data on cortical specification under Triple-i, we conclude that combined inhibition is indispensable for meaningful modelling of microcephaly development.

## Discussion

Methods for deriving cerebral organoids are highly diverse and give rise to immensely heterogeneous populations with respect to cortical identity. Despite this fact, comparative studies measuring the homogeneity of cortical fates are still exceptionally sparse. In this study we postulate that such cell-type heterogeneity may arise due to inherent non-cortical fate contamination present in the starting population and have therefore placed particular emphasis to reveal differences among NSCs derived using the different methods.

By systematically comparing methods side by side with constant reference to human in vivo databases, probing a number of developmental stages and generalizing our findings in hESCs and human iPSCs, we demonstrated differences between derivation methods and their respective regional biases. By employing bulk RNA-Seq, scRNA-Seq, NSC reporter line and immunostaining, we provided evidence that a short and early pathway modulation in NSC starting populations through combined Dual SMAD and WNT inhibition is critical for establishing long-lasting NSC cortical specification. This careful analysis has revealed that organoids generated by combined inhibition exhibit a highly consistent cortical NSC identity independent of the iPSC line.

We further provided evidence that early establishment of cortical NSC identity in organoids is pivotal for the generation of a richer cellular diversity at later stages of development. Specifically, using scRNA-Seq and immunostaining, we showed that day 50 organoids derived by combined inhibition selectively enrich for oRG cells populating well-defined oSVZ regions surrounding VZ areas. These VZ and iSVZ–oSVZ regions further develop (day 80) into discrete cortical units, which under Triple-i are enriched with oRG cells as well as deep and upper-layer neurons.

Our work also couples early cortical NSC homogeneity with robust radial organization within organoids. We demonstrate that only when derived by combined inhibition, NSCs marked by NOTCH activation (*HES5::eGFP* expression) and co-localized with cortical markers exhibit a strong capacity to radially organize (form rosettes). Rosettes have been documented for more than two decades, previously considered as an early intermediate of neural induction from PSCs^47^. In previous work, we isolated neural rosette-forming NSCs corresponding to early anterior neuroepithelial/radial glial-like cells from PSCs^4,5^ and in subsequent studies utilized NOTCH activation to prospectively isolate and characterize such neural rosettes as cortical VZ regions^7,48,49^. This study further advances this idea, showing that robustly forming NOTCH-active cortical rosettes are the primary mechanical groundwork critical for the establishment of cortical cell diversity through the generation of cortical units mirroring cortical germinal zone and neuronal layer development.

Finally, our study shows that the meaningfulness of disease phenotypes in organoid models is highly dependent on the derivation method. This study reveals that only when derived by combined inhibition, microcephaly organoids exhibit a significant reduction in size, dramatic loss in cortical gene expression and massive apoptosis within rosette regions. The overwhelming cortical identity of these rosettes suggests that the phenotypes recorded are cortex-specific. Given that developing human brains affected by microcephaly are inaccessible, it is nearly impossible to determine whether the laminar occurrence of CASP3 is an in vivo phenotype. However, case report studies investigating more than 100 cases of post-natal microcephaly identified a disproportionally large cerebellum compared with the cerebral cortex in 45% of the cases, suggesting involvement of genes that affect cerebral growth more than that of the cerebellum^50^. This is in agreement with the reduction of cortical and enrichment of hindbrain gene expression signatures in Triple-i organoids. Nonetheless, our data also show that other potential microcephaly phenotypes, such as increased differentiation and cell-cycle defects, can be observed in organoids generated by more than one method, implying that these phenotypes are either not cortex-specific or severe enough to be revealed in organoids with a lower cortical identity. Thus, a variety of derivation methods may be essential for assessing regionally specific pathophysiological aspects of microcephaly.

To conclude, the systematic comparison between different methods with respect to transcriptional profiles, cyto-architectural features and cell-fate acquisition has led to the identification of a short and early Dual SMAD-i and WNT-i method that substantially improves the limited cortical diversity in human organoids, thus recapitulating fundamental features of cortical development and offering a basis for organoid-based disease modelling.

## Methods

### Ethics declaration

For all data acquired using H9 hESCs, the entire experimental part was conducted and performed at Tel Aviv University, Israel, where research with commercially available hESCs such as H9 is not defined as human research and thus does not require approval from the ethics committee. For all data acquired using ZIP8K8 iPSCs, this work was overseen and approved by the ethics commission of the medical faculty of the Christian-Albrechts-Universität zu Kiel (project approval number A145/11).

### Generation and use of pluripotent cell lines

The BAC transgenic *HES5::eGFP* NOTCH-activation hESC reporter line was derived from the WA-09, XX (H9) hESC line (Wicell)^38^.

The microcephaly *STIL HES5::eGFP* NOTCH-activation reporter line was generated in our laboratory for this study as follows. We used clustered regularly interspaced short palindromic repeat–Cas9 genome editing to introduce a nonsense mutation at the *STIL* locus in the H9-derived *HES5::eGFP* hESC reporter line. This nonsense mutation mimics the microcephaly *STIL* mutation at amino-acid position 1218 (designated in this study as MC) in which deletion of the nucleotide G (at nucleotide position 3655) leads to a premature stop codon (Val1219X). A 21-nucleotide guide RNA (gRNA) sequence and a 150-nucleotide single-stranded DNA oligonucleotide (IDT; PAGE purified) were designed to target and generate double-strand breaks within the *STIL* ultimate exon (exon no. 17) upstream of the KEN domain and result in the generation of a truncation mutation via non-homologous end-joining and homology-directed repair. This gRNA was cloned into the pSpCas9 (BB)-2A-GFP (PX458, Addgene) plasmid downstream of the U6 promoter using the Gibson assembly cloning method. Human ESCs (400,000 cells) were nucleofected (Amaxa) with 1.5 μg of SpCas9 plasmid cloned with gRNA and 1 xμg of the single-stranded DNA. The cells were sorted for GFP signal by FACS 96 h after nucleofection. The sorted cells were replated at clonal density (5 × 10^3^ cells) on mouse embryonic fibroblasts (MEFs) supplemented with MEF-conditioned medium and 10 μM ROCK inhibitor with daily fresh FGF2. Individual colonies were manually picked and cultured in 24-well plates for a week and later expanded in six-well plates as individual clones. Genomic DNA was extracted from each clone and the targeted genomic region was sequenced. See Supplementary Table 1 for the *STIL* gRNA sequence, template sequence and primers.

The ZIP8K8 iPSC line (ZIP gGmbH) was derived from Human dermal fibroblast (HDF) cells obtained from a 40-year-old white male with no history of genetically inherited, neurological or metabolic disorders using a 3-mm punch biopsy. Briefly, the biopsy material was segmented into smaller fragments, plated onto tissue culture-treated plastic dishes and maintained in HDF medium containing DMEM Glutamax, 1% penicillin–streptomycin and 20% fetal calf serum at 37 °C and 5% CO_2_. The HDF medium was changed every alternate day to obtain a 90% confluent monolayer until the cells were passaged at a 1:3 ratio using trypsin. The PSC lines were induced using episomal plasmids following a published protocol, with minor modifications^51^. All plasmids were a gift from S. Yamanaka and obtained via Addgene (http://www.addgene.org). Briefly, 2 µg of the plasmids pCXLE-hSK (cat. no. 27080), pCXLE-hUL (cat. no. 27078) and pCXLE-hOCT3/4-shp53F (cat. no. 27077) were transfected into 1 × 10^6^ HDFs using the Neon microporator device with 100 µl electroporation tips (settings: 1.650 V, 10 ms, three timed pulses; Thermo Fisher Scientific) according to the manufacturer’s protocol. The transfected cells were resuspended in 10 ml fibroblast medium containing 90% 1×MEM (Thermo Fisher Scientific) supplemented with 10% fetal calf serum and the HDFs (2.5 × 10^4^ cells cm^−2^) were reseeded onto Matrigel-coated (0.5 mg ml^−1^; Corning) six-well plates. The fibroblast medium was replaced with TeSR-E7 (Stemcell Technologies) 2 d post transfection and the cells were fed every other day with 2 ml TeSR-E7 per well. On days 26–30 post transfection, the emerging iPSC colonies were picked and transferred onto Matrigel-coated (0.5 mg ml^−1^) plastic dishes in TeSR-E8 medium (Stemcell Technologies) for further expansion. Enzyme-free colony expansion using EDTA (0.5 mM; Invitrogen) was performed every 3–4 d at a 1:6 ratio as described previously^52^. Briefly, once the iPSC colonies reached 70–80% confluency, they were rinsed with 3 ml EDTA per dish and incubated with another 1 ml EDTA per dish for 2–5 min at room temperature. After incubation, the EDTA solution was removed and 1–4 ml of TeSR-E8 medium containing 10 µM ROCK inhibitor (Y-27632, Tocris) was used to wash the colonies off the plate and transfer them into a Matrigel-coated (0.5 mg ml^−1^) dish with fresh medium.

The additional iPSC lines used in this study include the human fibroblast-derived iPSC line KUCG2 (ref. ^53^; EBiSC, HPSI0214i-kucg_2), the human peripheral blood mononuclear cell-derived iPSC line FOK1 (hPSCReg, MPIPi008-A; a gift from M. Ziller, Max Planck Institute for Psychiatry, Munich), and the human fibroblast-derived iPSC line ZIP13K5 (ref. ^54^; ZIP gGmbH; provided by F.-J.M.).

### Culturing of undifferentiated PSCs

The H9-derived *HES5::eGFP* hESC and H9-derived *HES5::eGFP* microcephaly STIL*-*mutant hESC reporter lines were cultured on mitotically inactivated MEFs (Globalstem). The undifferentiated H9-derived hESC lines were maintained in DMEM/F12 medium containing 20% Knock-out Replacement medium (KSR), 1 mM glutamine, 1% penicillin–streptomycin, non-essential amino acids (100× solution; Thermo Fisher Scientific) and 50 µM β-mercaptoethanol (Thermo Fisher Scientific), and supplemented daily with 10 ng ml^−1^ FGF2 (R&D). The cells were passaged weekly using dispase (Worthington) to maintain their undifferentiated state. The human iPSC lines used in this study were cultured and maintained on Matrigel-coated (0.5 mg ml^−1^) dishes in mTesR1 medium (Stemcell Technologies) and passaged every 3–4 d using 0.5 mM EDTA to maintain their undifferentiated state.

### Neural induction and rosette formation from hESCs using the sEBs protocol

On day 0, hESC colonies were removed from the MEF feeder layer by exposure to 4 U ml^−1^ dispase (Worthington) and then dissociated using accutase (Innovative Cell Technologies, Inc.). Single hESCs were plated at a density of 750,000 cells per well in a six-well plate (low-attachment plate, Greiner) and cultured in 2 ml of neural induction medium—50% KSR medium (composed of knockout DMEM with 15% KSR, 2 mM glutamine, 1% penicillin–streptomycin, 1% non-essential amino acids and 50 µM β-mercaptoethanol (all from Thermo Fisher Scientific)) and 50% N2/Neurobasal medium (1:1). Our N2 medium (500 ml) was prepared as follows: 6 g DMEM/F12 powder (Thermo Fisher Scientific) supplemented with 0.775 g d-glucose, 1 g sodium bicarbonate, 12.5 mg insulin, 5 mg apo-transferrin, 30 µl of 500 µM sodium selenite, 60 µl of 830 nM putrescine, 100 µl of 100 µM progesterone and 1% penicillin–streptomycin (Sigma-Aldrich) in a total volume of 490 ml of milli-Q water. Neurobasal medium consists of Neurobasal medium (Thermo Fisher Scientific) as the base supplemented with 2 mM glutamine, 1% penicillin–streptomycin, 1% non-essential amino acids and 50 µM β-mercaptoethanol (Thermo Fisher Scientific). We refer to this Neurobasal medium with supplements as NB from hereon. The final prepared medium was also supplemented with 1% B27 without retinoic acid (RA; Invitrogen) and 10 µM ROCK inhibitor. Small embryoid bodies (sEBs) were allowed to form for 2 d. On day 2, the medium was changed to 25% KSR medium plus 75% N2/NB medium supplemented with B27 without RA and 10 µM ROCK inhibitor. In addition, the sEBs were either untreated (Inhibitor-free) or treated with 10 µM SB-431542 (Tocris) plus 250 ng ml^−1^ NOG (R&D; Dual SMAD-i), 3.3 µM XAV-939 (1:6,000 from a 20 mM stock; WNT-i) or all three in combination (Triple-i). The sEBs were collected on day 3 and plated on culture dishes pre-coated with 15 μg ml^−1^ polyornithine (Sigma), 1μg ml^−1^ laminin and 1 μg ml^−1^ fibronectin (BD Biosciences). The medium and factors were complemented as required, with the same composition as that for day 2, except for ROCK inhibitor, and the medium was left unchanged for the next 4 d. On day 7, the medium was changed to 100% N2/NB medium supplemented with 1% B27 without RA and further supplemented with 5 µM SB-431542, 125 ng ml^−1^ NOG and 3.3 µM WNT-i. On day 9, the medium was replaced, and the inhibitors were withdrawn and replaced by 100 ng ml^−1^ FGF8 and 5 ng ml^−1^ BDNF (R&D). Rosettes were allowed to form until day 12, following which the cells were fixed, harvested for analysis or subjected to terminal differentiation. Neural induction and direct rosette formation could also be obtained by adherence on Matrigel-coated plates as described in Edri et al^7^., with the modifications defined for this study (such as addition of XAV-939 and so on). Briefly, hESC colonies were removed from MEFs with 6 U ml^−1^ dispase and dissociated with accutase. The cells were then plated at subconfluent cell density (40–50 × 10^3^ cells cm^−2^) on Matrigel-coated dishes (1:20; Corning) containing MEF-conditioned medium with 10 μM ROCK inhibitor and further supplemented by daily addition of 10 ng ml^−1^ FGF2 (R&D). Confluent cultures were subjected to the appropriate neural differentiation treatment and, on day 10–12, neuroepithelial (NE) cells were incubated with Ca^2+^ and Mg^2+^-free HBSS solution, followed by 2.5 mg ml^−1^ collagenase II, 2.5 mg ml^−1^ collagenase IV and 0.5 mg ml^−1^ DNase (all from Worthington) solution for 20 min at 37 °C. After incubation, the cells were scrapped from the plates and subsequently dissociated and replated at high density (5 × 10^5^ cells cm^−2^) on moist Matrigel drops. Long-term propagation of cortical neural progenitors was performed weekly by manually picking rosettes, followed by re-plating on polyornithine, laminin and fibronectin-coated dishes and adding 100% N2/NB medium containing 1% B27 without RA plus either FGF8 and BDNF (until day 28) or 20 ng ml^−1^ of FGF2, EGF and BDNF (R&D; from day 28 onwards). The same protocol was used for the human ZIP8K8 iPSC line, except that undifferentiated cells were first treated with EDTA or trypLE (instead of dispase used for hESCs) and then dissociated using accutase. Single cells were then plated under the same differentiation conditions used for H9 hESCs.

### Derivation and analysis of cerebral organoids

On day 0, *HES5::eGFP* hESC colonies were first incubated with 1 ml dispase (4 U ml^−1^) for 7–10 min at 37 °C in an incubator until colonies detached from feeder cells (MEFs). Human ESC medium (as described earlier in the ‘Culturing undifferentiated PSCs’ section) was used to neutralize the dispase; the colonies were washed twice with hESC medium and allowed to sink to the bottom of the falcon tube. The supernatant was aspirated, 1 ml accutase was added along with ROCK inhibitor (10 µM) and the colonies were kept in a water bath (37 °C) for 4 min. The colonies were then triturated 15 times using a p1000 tip until single cells were obtained. The accutase enzyme was neutralized by washing twice with hESC medium and centrifugation at 270*g* for 5 min. Single cells were resuspended in 1 ml hESC medium containing FGF2 (4 ng ml^−1^) and ROCK inhibitor (50 µM; Tocris). The cells were enumerated and the volume of the hESC medium was adjusted along with FGF2 and ROCK inhibitor to a concentration of 9,000 cells per 150 µl. For the iPSC lines, a feeder-free culture system was used where cells were first incubated with 1 ml EDTA (0.5 mM) for 2 min at 37 °C in the incubator, after which the EDTA was substituted with 1 ml accutase (per 60-mm culture dish) and the cells were incubated for 3 min at 37 °C. The cells were then triturated 10–15 times using p1000 tips until single cells were obtained. The single-cell suspension was first washed with mTesR1 and then with hESC medium containing FGF2, after which the cells were centrifuged at 270*g* for 5 min. Single cells were resuspended, counted and plated similarly as for hESCs. Suspended single cells (9,000 per 150 µl) were plated on a 96-well U-bottom low-attachment plate (Corning). The plate was inspected for cell aggregation and formation of sEBs on day 1. On day 2, half of the medium was aspirated without disturbing aggregates and 150 µl hESC medium was added to a total of 225 µl hESC medium along with the appropriate inhibitor molecule—SB-431542 (10 µM), NOG (250 ng ml^−1^) or XAV-939 (3.3 µM; 1:6,000 from a 20 mM stock)—or a combination thereof. FGF2 and ROCK inhibitor were withdrawn once the sEBs reached a size of approximately 350 µm. On day 4, 150 µl medium was removed and replaced with fresh 150 µl hESC medium along with the corresponding inhibitor molecules. On day 6, the organoids were transferred into a low-attachment 24-well plate along with N2 neural induction medium (composition as described in the ‘Neural induction and rosette formation from hESCs using the sEBs protocol’ section). Every alternate day, 300 µl medium was aspirated and replaced by an equal volume of fresh N2 medium along with factors until day 11. On day 11, the organoids (500–600 µm in size) were embedded in 30 µl Matrigel droplets and incubated for 30 min in the incubator, after which they were transferred into a six-well low-attachment plate containing N2/NB medium along with 1% B27 without RA using a sterile spatula. On day 13, a medium change was made using the same medium from day 11. On day 15, the entire supernatant medium was removed and replaced with fresh medium containing N2/NB medium along with 1% B27 with RA; the organoid dishes were transferred onto an orbital shaker and the medium was changed daily. For long-term organoid culture, Matrigel (1%) was added directly to the culture medium and the medium was changed every 2 d. Organoids were fixed in 4% paraformaldehyde for 20–40 min (room temperature) depending on their culture age, and then cryoprotected and processed as described under the ‘Immunostaining and confocal imaging’ section.

A step-by-step protocol for the generation of cerebral organoids with enriched cortical cellular diversity is available at *Protocol Exchange*^55^.

### Preparation and sequencing of bulk RNA-Seq libraries

For all H9- and ZIP8K8-derived organoids, RNA was purified using an miRNeasy RNA MiniPrep kit (Qiagen). RNA-Seq libraries were generated for H9- and ZIP8K8-derived organoids (Dual SMAD-i (*n* = 1; five organoids, pooled), Inhibitor-free (*n* = 1; five organoids, pooled), WNT-i (*n* = 1; four organoids, pooled), TGFB and WNT-i (*n* = 1; four organoids, pooled) and Triple-i (*n* = 1; five organoids, pooled)) using Illumina TruSeq RNA library preparation kits and sequenced on an Illumina HiSeq 2500 sequencer as 100-bp and 76-bp paired-end reads, respectively. For Triple-i ZIP8K8-derived organoids (*n* = 5; four organoids, pooled), RNA-Seq libraries were generated using a NEBnext UltraDirectional RNA library preparation kit after ribosomal RNA depletion using a NEBNext rRNA depletion kit and sequenced on an Illumina HiSeq 2500 sequencer using 50 cycles of single-end sequencing.

### Description of processed RNA-Seq datasets of human brain transcriptomes

Gene expression data for different brain regions were retrieved from the BrainSpan Atlas of the Developing Human Brain (http://human.brain-map.org/) based on an extensive RNA-Seq study conducted by Šestan and colleagues^34^. Of the samples collected for that study, for our analysis we utilized datasets obtained from 16 brain regions (dissected at weeks 8–37 of gestation), 11 of which were obtained from different neocortical regions and the remaining five were collected from the hippocampal primordia (future hippocampus), sub-cerebral regions including the diencephalon (future thalamic structures) and the sub-pallium (future striatum) as well as posterior brain regions (cerebellum). The file ‘RNA-Seq Gencode v10 summarized to genes’ containing reads per kilobase of transcript per million mapped reads (RPKM) values (available at http://www.brainspan.org/static/download.html) was downloaded on 3 August 2017. A detailed description of the data processing procedures for generating the above file by the authors is available at http://help.brain-map.org/display/devhumanbrain/Documentation.

### RNA-Seq data processing and normalization for single and pooled organoids

For H9-derived organoids, raw RNA-Seq reads were mapped to the human reference genome hg19 using STAR mapper version 2.6.1d^56^. The generated bam files were filtered for uniquely mapped reads using Samtools version 1.10 (ref. ^57^) and read counts were generated using HTSeq version 0.10.0 (ref. ^58^; parameters: -m intersection-strict–nonunique all -r pos -s reverse). For further analysis, all mitochondrial genes were removed from the data. The RPKM values were calculated by dividing the number of counts by the gene length and sequencing depth, and then multiplying by 10^9^. For data normalization, only RefSeq-annotated genes were considered and their RPKM values were normalized by bringing the samples to the same RPKM sum. The normalized RPKM values for the 3D (organoid) samples are summarized in Supplementary Table 2. For generating heatmaps, a pseudocount of one was added to the RPKM values, which were then log_2_-transformed. All rows (genes) of the heatmap matrices were then scaled to a range of −0.5 to 0.5.

For ZIP8K8-derived organoids, reads were first trimmed using Trimmomatic^59^ (version 0.36; parameters: leading, 3; trailing, 3; slidingwindow, 4:15; minlen, 36). The trimmed reads were then aligned to the human reference genome hg19 using STAR mapper version 2.6.1d^56^ and Gencode v19 gene annotations (https://www.gencodegenes.org/human/release_19.html). Read counts and FPKM values were then estimated using RSEM version 1.3.1 (ref. ^60^) with the command ‘rsem-calculate-expression’. The log_2_-transformed fold-change values were then calculated from the averaged log_2_-transformed FPKM values after adding a pseudocount of one for each protocol. The fold changes of Allen brain regional genes (see the ‘Differential gene expression and gene-set enrichment analysis between organoids’ section for Allen brain regional gene set estimation) are shown in Extended Data Fig. 1e. The FPKM values are included in Supplementary Table 3.

### Combined RNA-Seq data analysis for organoid and human brain datasets

As raw RNA-Seq datasets for human brain regions were accessible, comparative analysis of BrainSpan and cerebral organoid samples together was reprocessed to minimize the processing differences between both datasets. This was achieved first by reprocessing organoid datasets as described in the previous section for processing organoid datasets alone but with the HTSeq parameter -s changed to ‘no’. In addition, all values generated by merging both RPKM matrices were quantile normalized. To remove non-biological variations revealed by the correspondence analysis (see the next section), the function ComBat^61^ from the sva package was applied. ComBat uses an empirical Bayesian framework to adjust data for batch effects and other unmeasured sources of variation. ComBat-transformed RPKM values are shown in Supplementary Table 4.

### Correspondence analysis

Correspondence analysis^62^ is a projection method that represents variables such as expression values of genes as vectors in a multidimensional space. Similar to principal component analysis (PCA), correspondence analysis also reveals principal axes of the investigated space. This allows projection of the data matrix into a low-dimensional subspace and hence investigation of the main variance in the data. Moreover, in contrast to PCA, correspondence analysis can simultaneously account for samples in a gene-dimensional space and genes in a sample-dimensional space, showing the information in a so-called biplot. The interpretation of correspondence analysis biplot is such that one finds the genes characteristic for a (group of) sample(s) in the direction of this sample (group). The further away from the centre the genes lie, the more characteristic they are of the respective sample(s).

Both correspondence analyses from our study were conducted using the 10,000 genes with the highest expression variance across the investigated samples. In the combined analysis of brain and organoid samples, we first merged both RPKM matrices and projected the new matrix into the 3D subspace. The resulting correspondence analysis plot shows very clearly that the first principal axis accounts for the technical variation between both datasets (data not shown). Hence, to remove the observed bias we applied ComBat; the result of the final correspondence analysis of the ComBat-transformed data is shown in Fig. 1b,c.

### Differential gene expression and gene-set enrichment analysis between organoids

From the Allen Human Brain Atlas dataset, we estimated markers for different brain regions during weeks 12–21 by comparing the log_2_-transformed fold change of the RPKM expression value for each regional sample compared with all other regions across weeks 12–21. Genes were defined as regionally specific if they had a log-transformed fold-change value of at least two when compared with the samples from all other regions, excluding striatal and amygdala samples, across all weeks. We furthermore filtered striatal- and amygdala-specific genes by removing genes with a log-transformed fold-change value of two in amygdala or striatal samples compared with all other regions across weeks 12–21. To determine differential gene expression across brain organoids derived using different protocols, we ran DeSeq2 (ref. ^63^) on the count data across three pairwise treatment comparisons—Triple-i versus Dual SMAD-i, Triple-i versus Inhibitor-free and Dual SMAD-i versus Inhibitor-free—using eight biological replicates of individual organoids from each protocol. A gene-set enrichment analysis^64^ was then run to determine the significance of enrichment of the regional specific gene sets in each of the three comparisons. Finally, we estimated the in vivo relative expression levels of protocol-specific and shared regional genes. First, the summed expression of genes from each regional gene set across region-specific BrainSpain samples (that is, for cortical genes, expression level across all cortical samples) from weeks 12–21 was estimated. Next, the average of these summed expressions was calculated for regional genes forming each category of protocol comparison (that is, protocol-specific regional genes that were consistently upregulated in each pairwise comparison and shared genes that were not significantly up- or downregulated in any pairwise comparison). Finally, a *z*-score was estimated from these averages across categories with a minimum of five genes and plotted in the Venn diagrams in Fig. 1g. The DeSeq2 results have been deposited at Gene Expression Omnibus under the accession number GSE189981. Regional gene sets are included in Supplementary Table 5.

### Differential gene expression and pathway enrichment across microcephaly organoids

A differential gene expression analysis was conducted using DESeq2 on the raw-count data to compare day 17 and day 30 heterozygous and homozygous microcephaly pooled organoids versus wild-type pooled organoids in the Triple-i, Dual SMAD-i and Inhibitor-free organoid groups. The log_2_-transformed fold changes of Allen Human Brain Atlas regional genes in day 30 organoids derived from DESeq2 are shown in Fig. 8d. The DESeq2 analyses have been deposited at Gene Expression Omnibus under the accession number GSE189981.

### scRNA-Seq procedures

#### Organoid dissociation

Day 50 organoids (*n* = 4 or 5 pooled organoids per sample) derived by Triple-i and Dual SMAD-i (from ZIP8K8, ZIP13K5, KUCG2 and FOK1 iPSC cell lines), and Inhibitor-free (from ZIP8K8 and ZIP13K5 cell lines) treatments; individual day 80 organoids derived by Triple-i (ZIP8K8 cell line; *n* = 3 organoids) and Dual SMAD-i (ZIP8K8 cell line; *n* = 3 organoids) as well as pooled Triple-i organoids (ZIP13K5 cell line; one pooled experiment across *n* = 3 organoids) were dissociated into single cells using a papain dissociation kit (Worthington). The organoids were dissected into small pieces, incubated with papain and DNase I solution for 35–45 min, triturated and the cell suspension was filtered twice through 40-µm filter to obtain a single-cell suspension. The cells were centrifuged at 300*g* for 5 min, resuspended in Dulbecco’s phosphate buffer solution containing 0.4% BSA and counted for viability (>80%).

### Single-cell library preparation

Roughly 17,400 single live cells (1,000 cells µl^−1^) in Dulbecco’s phosphate buffer solution containing 0.4% BSA were used for Gel Beads-in-emulsion (GEM) generation, barcoding and library preparation according to the manufacturer’s recommendations for the 10X Chromium single cell 3′ reagent kit v3.1. For the day 50 organoids, nine cycles were used for complementary DNA amplification, whereas 12 cycles were performed for library construction. For the day 80 organoids, 11 cycles were used both for cDNA amplification and library construction. The resulting libraries were sequenced using Illumina short read sequencing.

### Processing and analysis of day 50 scRNA-Seq data

The scRNA-Seq data from day 50 organoids were processed using the Cell Ranger software version 3.1.0 (ref. ^65^) reference genome hg38 and ensembl reference transcriptome version 93 (http://ftp.ensembl.org/pub/release-93/gtf/homo_sapiens/Homo_sapiens.GRCh38.93.gtf.gz). Cell barcodes that had at least 10,000 unique molecular identifiers (UMIs) or at least 40% mitochondrial UMIs were filtered from the downstream analyses. The raw-count matrices from single cells across all day 50 organoids were then loaded into scanpy version 1.5.1. The count data were normalized such that each cell had a total count equal to the median of the total counts before normalization using scanpy’s pp.normalize_total function. The natural logarithm of these normalized counts was then calculated after adding a pseudocount of one using the log1p function in numpy. The top 2,000 highly variable genes with a mean normalized expression value between 0.005 and 1.5 were then calculated using the pp.highly_variable_genes function in scanpy, and subsequent dimension reduction and clustering was applied to the log-normalized data after subsetting to these genes. First, a PCA was applied. A neighbourhood graph of observations was then constructed from the top 50 principal components using scanpy’s pp.neighbours function with n_neighbors = 14. A UMAP embedding was then estimated from the neighbourhood graph using the parameters min_dist = 0.1 and spread = 1. The UMAP is shown in Fig. 2a–e. Clusters were then estimated using the Louvain method for community detection^66^ in scanpy with resolution = 4.

Doublets were detected by running scrublet version 0.2.3 (ref. ^67^) on each sample separately with the input parameter expected_doublet_rate = 0.05 and applying a doublet score threshold of 0.2. More than 40% of the cells in Cluster 40 were annotated as doublets by scrublet, whereas all other clusters contained 1–12% doublets. Therefore, all cells assigned as doublets using scrublet, along with all cells from cluster 40, were annotated as doublets and subsequently removed from further analyses and plotting. The remaining clusters were manually assigned to a cell type and region based on the differential expression of well-established marker genes. Metadata for all 96,454 cells is in Supplementary Table 6.

Differential expression of genes per cluster was estimated using a *t*-test with scanpy’s rank_genes_group function and the method t-test_overestim_var. Genes were annotated as significantly differentially expressed within a cluster if they had a log_2_-transformed fold-change value of at least one and a *q*-value less than 0.05 after applying a Benjamini–Hochberg multiple hypothesis correction to the estimated *P* values. Clusters 24 and 31 were annotated as ‘unknown’ due to the lack of known differentially expressed marker genes in these clusters. Differential gene expression analysis for each cluster is in Supplementary Table 7.

### Merging the day 50 organoid and Bhaduri scRNA-Seq datasets

We first subset cells collected at weeks 8 and 10 of in vitro development from Bhaduri et al.^31^ and then processed the scRNA-Seq data in a similar manner as described in their paper. The log-normalized count data were loaded into scanpy. Batch, indicated in the metadata, was regressed out using scanpy’s regress_out function. The expression was then scaled to have unit variance and zero mean, and values were truncated to a maximum value of ten. The log-transformed expression values for cells from our organoids were similarly scaled and truncated to a maximum value of ten. The top 2,000 highly variable genes with a mean normalized expression value of at least 0.0125 were then estimated from the log-normalized expression data in our organoids and separately across the organoids in^31^. We then subset to the union of highly variable genes across both datasets and applied a PCA to the data. A neighbourhood graph was constructed from the top 50 principal components using a batch-balanced *k*-nearest neighbour graph approach^68^ to account for batch differences across the studies. Finally, a UMAP embedding was estimated from the batch-corrected neighbourhood graph with the parameters min_dist = 0.1 and spread = 1. The UMAP is shown in Extended Data Fig. 4a, with cells coloured according to the derivation protocol, and the scaled expression values of *FOXG1* are shown in Extended Data Fig. 4b. We then measured Pearson’s correlation between the average scaled expression levels across all cells within each of our organoid clusters and the Bhaduri organoid clusters after subsetting to the union of highly variable genes across both datasets. The Pearson’s correlation values are in Extended Data Fig. 4c. Individual clusters from the Bhaduri organoids were then assigned to specific regions based on the differential expression of well-established brain-region gene sets. Pearson’s correlations between our cortical organoid clusters and the Bhaduri in vivo clusters across cells from all developmental weeks were estimated in the same way as described above for the Bhaduri in vitro clusters and are shown in Fig. 4c.

### Differential gene expression of day 50 cortical cell types

We ran a differential gene expression analysis comparing dividing and non-dividing cortical NSCs (clusters 29, 4, 15, 0, 19, 27 and 12) in Triple-i and Dual SMAD-i organoids. Genes were tested if they were expressed in at least 1% of all cortical NSCs from either protocol. Differential expression was estimated using a *t*-test with scanpy’s rank_genes_group function and the method t-test_overestim_var. Genes were annotated as significantly differentially expressed within a protocol if they had an absolute log_2_-transformed fold-change value of at least one and a *q*-value less than 0.05 after applying a Benjamini–Hochberg multiple hypothesis correction to the estimated *P* values. We then highlighted oRG-specific genes from Pollen and colleagues^41^ (66 genes in total) that were significantly differentially expressed in Fig. 4d.

Next, we ran a differential gene expression analysis comparing cortical clusters within Triple-i and Dual SMAD-i organoids separately. Differential expression was again estimated using a *t*-test with scanpy’s rank_genes_group function and the method t-test_overestim_var. Only genes expressed in at least 2% of cells within at least one cluster were tested, and they were labelled as significantly upregulated if they had a log_2_-transformed fold change of at least one and *q*-value less than 0.05 after applying a Benjamini–Hochberg multiple hypothesis correction to the estimated *P* values.

### Processing and analysis of day 80 scRNA-Seq data

The scRNA-Seq data from day 80 Triple-i and Dual SMAD-i organoids were processed using the Cell Ranger software (version 6.0.1) using the same reference genome as day 50 organoids. Cell barcodes that had at least 10,000 UMIs, at least 20% mitochondrial UMIs or at least 15% ribosomal UMIs were filtered from downstream analyses, which were conducted in scanpy version 1.5.1. Similar to day 50 organoids, doublets were detected by running scrublet version 0.2.3 (ref. ^67^) on each sample separately with the input parameter expected_doublet_rate = 0.05 and applying a doublet score threshold of 0.2. All estimated doublets were removed from further downstream analyses. The count data were then normalized using the pp.normalize_total function in scanpy, after which a log1p normalization was applied. The top 2,000 highly variable genes with a mean normalized expression value between 0.005 and 1.5 were then calculated using the pp.highly_variable_genes function in scanpy. The expression values of these genes were then scaled to have unit variance and zero mean, and the values were truncated to a maximum value of ten. To remove batch effects, we employed scanorama^69^, a panoramic stitching algorithm, using the scaled expression values as input and treating each individual organoid as its own batch. The top 20 dimensions from the scanorama batch-corrected low-dimensional embedding was then used to build a *k*-nearest neighbours graph with n_neighbors = 20. A UMAP embedding was then estimated from the neighbourhood graph using the parameters min_dist = 0.6 and spread = 1, and is shown in Fig. 7b. Clusters were then estimated using the Louvain method for community detection in scanpy with resolution = 2. The clusters were manually assigned to a cell type and region based on the differential expression of well-established marker genes, including deep- and upper-layer neuronal marker gene sets^44,45^. Metadata for all 65,670 cells is in Supplementary Table 8. Differential gene expression analysis for each cluster is in Supplementary Table 9.

### Day 80 scRNA-Seq RNA velocity

We ran velocyto version 0.17.16 (ref. ^70^) on the day 80 scRNA-Seq data to first measure the number of spliced and unspliced UMIs for each gene in each cell. We next loaded these spliced and unspliced counts into Python using the scvelo package version 0.1.24 (ref.^71^). We then removed mesenchymal cells as these cells were not part of the neural lineage. Genes with fewer than 30 total spliced counts and fewer than 30 total unspliced counts across all cells in the dataset were filtered, leaving 8,982 genes. The spliced and unspliced counts were normalized separately such that each cell had a total count equal to the median of total counts before normalization, after which a log1p transformation was performed. After this, the spliced and unspliced counts were averaged over the 30 nearest neighbours of each cell using the pp.moments function in scvelo, in effect smoothing the data across the local neighbourhood of each cell. Finally, RNA velocity was estimated using the ‘deterministic’ mode with the scvelo tl.velocity function, which measures the RNA velocity as the deviation from the approximated steady-state equilibrium over the locally averaged spliced and unspliced counts for each cell. The velocity estimates were then embedded onto the UMAP after removing mesenchymal cells using the tl.velocity_graph and pl.velocity_embedding_stream functions in scvelo.

### Differential gene expression of day 80 cortical NSCs

We ran a differential gene expression analysis comparing cortical NSCs in ZIP8K8 and ZIP13K5 Triple-i and ZIP8K8 Dual SMAD-i organoids. Genes were tested if they were expressed in at least 1% of all cortical NSCs from either protocol. Differential expression was estimated using a *t*-test with scanpy’s rank_genes_group function and the method t-test_overestim_var. Genes were annotated as significantly differentially expressed within a protocol if they had an absolute log_2_-transformed fold change of at least 0.1 and *q*-value less than 0.1 after applying a Benjamini–Hochberg multiple hypothesis correction to the estimated *P* values. We then ran a two-sided Fisher’s exact test to estimate the enrichment of oRG-specific genes derived from Pollen et al.^41^ among the upregulated genes in Triple-i (*P* = 1.2 × 10^−10^) and separately among the upregulated genes in Dual SMAD-i (*P* = 0.1).

### Immunostaining and confocal imaging

Cells were fixed in 4% paraformaldehyde and 0.15% picric acid, and permeabilized and blocked with PBS containing 1% BSA, 10% FBS and 0.3% Triton solution. Organoids were similarly fixed, washed, cryoprotected with 30% sucrose overnight and then submerged in optimal cutting temperature compound. Fixed cells or sectioned organoids (10-µm slices) were stained with the indicated primary antibodies (see the next section), followed by Alexa Fluor secondary antibodies (Invitrogen). Following staining, the cells were imaged in PBS and the organoid sections were mounted on Moviol (Sigma). Fluorescence images were obtained using an LSM710 confocal microscope (Carl Zeiss Micro Imaging). The confocal images were captured using ×10 and ×20 objectives (numerical aperture = 0.3 and 0.8, Plan-Apochromat, respectively). Fluorescence emissions resulting from Ar 488-, 543- and 633-nm laser lines for EGFP, CY3 and CY5, respectively, were detected using the laser scanning settings and filter sets supplied by the manufacturer. For DAPI detection in all images as well as GFP detection for organoid images, we used our mode-locked Ti:Sapphire, fento second pulsed, multiphoton laser (Chameleon Ultra II, Coherent, Inc.) at a wavelength of 720 and 920 nm, respectively. Epifluorescent and phase-contrast images were obtained using a Nikon Eclipse Ti-E microscope. Fluorescence emissions results from mercury arc lamp. Images were taken using ×10 and ×20 objectives. Images were generated and analysed using either the Zeiss ZEN 2011 (Carl Zeiss, Inc.) or NIS-elements (Nikon) software. All images were exported in TIF format and their colour levels were identically adjusted for each staining procedure.

### Antibody list

The antibodies to EMX2 (ab94713; 1:50), FOXG1 (ab18259; 1:400), p-VIM (ab22651; 1:120), SOX2 (ab79351; 1:500), SATB2 (ab51502; 1:50), CTIP2 (ab18465; 1:250), TBR1 (ab31940; 1:500) and CUX1 (ab54583; 1:200) were from Abcam. The antibodies to OCT3/4 (sc5279; 1:22) and LIFR (sc-515337; 1:100) were from Santa Cruz. The antibody to PAX6 (supernatant, 1:22) was from DSHB. The antibodies to SOX1 (AF3369; 1:40), SOX2 (AF2018; 1:100) and OLIG3 (MAB32456; 1:450) were from R&D. The antibodies to NR2F1 (ABE1425; 1:500), DCX (AB2253; 1:500) and LMX1A (AB10533; 1:1,000) were from Millipore. The antibodies to SP8 (HPA054006; 1:50) and MEF2C (HPA005533; 1:100) were from Atlas Antibodies. The antibodies to CASP3 (cat. no. 9661; 1:500) and TCF7L2 (cat. no. 2569; 1:500) were from Cell Signaling. The antibody to TTR (AHP1837; 1:500) was from Bio-Rad. The antibodies to HOPX (HPA030180; 1:500), PTPRZ1 (HPA015103; 1:500) and EMX1 (HPA006421; 1:50) were from Sigma-Aldrich. Secondary Alexa Fluor antibodies 488, 546 and 647 (1:700) were obtained from Invitrogen.

### Quantitative PCR analysis

RNA was extracted using an miRNeasy kit (Qiagen), followed by transcription using a cDNA reverse transcription kit (Applied Biosystems). The cDNA (4–6 ng) was subjected to quantitative PCR using our homemade designed primers (listed in ‘Primer set list’), FastStart universal SYBR green (Roche) and ViiA-7cycler (ABI). Threshold cycle values were determined in triplicate and presented as the average fold change relative to *HPRT*. The fold changes were calculated using the $2^{{-{\Delta}C}_{\rm{t}}}$ method.

### Primer set list

The following primers were used: BRACHYURY forward, 5′-ACCCAGTTCATAGCGGTGAC-3′ and reverse 5′-CAATTGTCATGGGATTGCAG-3′; FOXA2 forward, 5′-CCGACTGGAGCAGCTACTATG-3′ and reverse 5′-TGTACGTGTTCATGCCGTTC-3′; HPRT forward, 5′-TGACACTGGCAAAACAATGCA-3′ and reverse 5′-GGTCCTTTTCACCAGCAAGCT-3′; OCT4 forward, 5′-CAGCAGATCAGCCACATC-3′ and reverse 5′-CGGTTACAGAACCACACTC-3′; SOX1 forward, 5′-GCAAGATGGCCCAGGAGAAC-3′ and reverse 5′-CGGACATGACCTTCCACTCG-3′; SOX17 forward, 5′-AAGATGACTGGCAAGTCGT-3′ and reverse 5′-CTTCAGCCGCTTCACCTG-3′; and SOX2 forward, 5′-GCAAGATGGCCCAGGAGAAC-3′ and reverse 5′-CCGACAAAAGTTTCCACTCGG-3′.

### Image analysis

Immunostainings for regional markers in Fig. 2 and cortical neuronal markers in Fig. 6 were processed and analysed on a dedicated Zen Blue version 3.2 workstation (Zeiss). Briefly, *z*-stacked images underwent maximal intensity projection and tiled images were stitched together before proceeding with quantification. Image analysis was performed using the Image analysis module in the Zen software suite, and followed a hierarchical strategy. The full images or image subsets were divided into two segregating classes (rosettes versus non-rosettes or cortical units versus non-cortical units) by taking advantage of the locally enhanced density of nuclei. Therefore, thresholds were determined from fixed fluorescence intensity values from over-smoothed nuclear counterstain images. Within the two resulting classes, single cells were identified by faint smoothing, rolling-ball background subtraction, watershedding and fixed intensity thresholds. The resulting masks were applied to the raw input images and the fluorescence intensity for all channels were calculated within regions of interest as the mean intensity. Marker intensity cutoffs were determined manually to classify positive cells and the results were plotted in custom Python scripts.

### Statistics and reproducibility

For all quantitative qPCR gene expression analysis for the iPSC characterization experiments, a two-way analysis of variance test, followed by Tukey’s multiple comparison test was applied. For calculating organoid size, the ImageJ or NIS-elements (Nikon) software was used. Statistical analyses were performed using the GraphPad software and the scipy.stats package in Python 3.

The exact number of organoids in the immunostaining experiments are indicated in the respective figure legends (Figs. 1a, 2, 5, 6, 7a, 8a–c,e–h and Extended Data Figs. 1e, 2, 5–8, 9e,f) and all numerical values used for the quantification are detailed in the respective Source Data files. The statistical tests used in each figure are detailed in the figure legend and *P* values are presented in the respective figures. No statistical method was used to pre-determine sample size. Experiments in this work were not randomized and no blinding was used during the data analyses.

### Reporting summary

Further information on research design is available in the Nature Research Reporting Summary linked to this article.

## Online content

Any methods, additional references, Nature Research reporting summaries, source data, extended data, supplementary information, acknowledgements, peer review information; details of author contributions and competing interests; and statements of data and code availability are available at 10.1038/s41556-022-00929-5.

## Supplementary information

Supplementary Information Supplementary Fig. 1. ZIP8K8 iPSC line characterization.

Reporting Summary

Supplementary Table 1 Supplementary Tables 1–9.

Supplementary Data 1 Quantitative PCR STEMdiff expression levels for ZIP8K8 cells relative to *HPRT*.

## Data Availability

The RNA-seq datasets derived from H9, ZIP13K5 and KUCG2 cell lines have been deposited in the Gene Expression Omnibus under the accession code of GSE189981. The RNA-seq datasets derived from the cell lines ZIP8K8 and FOK1 have been deposited in the European Genome-Phenome Archive under the accession code of EGAS00001006063. Previously published scRNA-Seq data that were re-analysed here are available in the Gene Expression Omnibus under the accession code GSE132672. Previously published bulk RNA-Seq data that were re-analysed here from the BrainSpan Atlas of the Developing Human Brain are available at https://www.brainspan.org/static/download.html under ‘RNA-Seq Gencode v10 summarized to genes’. All other data supporting the findings of this study are available from the corresponding author on reasonable request. Source data are provided with this paper.

## Source data

Source Data Fig. 2 Mean *HES5::eGFP* intensity levels and number of *HES5::eGFP-*positive rosettes in day 17 organoids. Immunostaining counts of regional markers, rosette numbers.
Source Data Fig. 5 Immunostaining counts of iSVZ/oSVZ markers in day 50 ZIP8K8-, ZIP13K5-, KUCG2- and FOK1-derived organoids.
Source Data Fig. 6 VZ areas and immunostaining counts of oSVZ markers outside VZ areas across cortical units in day 80 ZIP8K8-derived organoids.
Source Data Fig. 7 Immunostaining counts of upper- and deep-layer neuronal markers across cortical units in day 80 ZIP8K8-derived organoids.
Source Data Fig. 8 Day 17 WT and MC organoid sizes. Immunostaining counts of CASP3 in day 20 WT and MC organoids.
Source Data Extended Data Fig. 2 Immunostaining counts of NSC and cortical markers in day 12 monolayer cultures. Immunostaining counts of cortical markers in day 30 ZIP8K8-derived organoids.
Source Data Extended Data Fig. 9 SOX2/DCX areas, p-VIM nuclei per vesicle area measured from immunostainings of day 20 WT and MC organoids.

## Code availability

The code used for the data analyses is available on GitHub at https://github.com/daniel-rosebrock/BrainOrganoids.
