# Supplementary material for: Enhanced cortical neural stem cell identity through short SMAD and WNT inhibition in human cerebral organoids facilitates emergence of outer radial glial cells
Source: Nat Cell Biol. 2022 Jun 13;24(6):981–95. doi: 10.1038/s41556-022-00929-5 (PMC9203281; doi:10.1038/s41556-022-00929-5)
Supplement: Supplementary file 1 — Supplementary Fig. 1. ZIP8K8 iPSC line characterization. [file 41556_2022_929_MOESM1_ESM.pdf]

nature portfolio

https://doi.org/10.1038/s41556-022-00929-5

Supplementary information

Enhanced cortical neural stem cell identity through short SMAD and WNT inhibition in human cerebral organoids facilitates emergence of outer radial glial cells

In the format provided by the authors and unedited

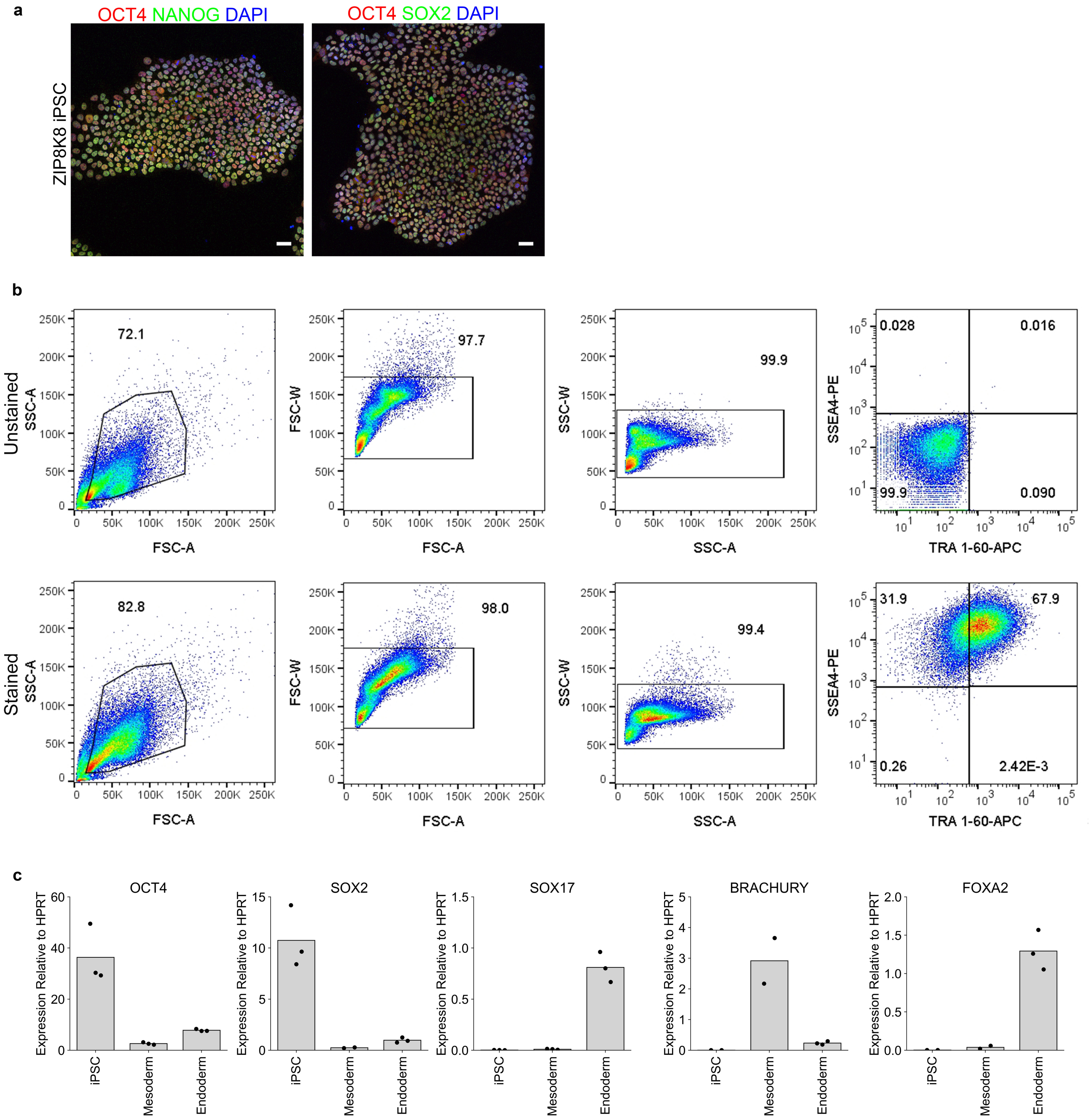
a
OCT4 NANOG DAPI
OCT4 SOX2 DAPI
ZIP8K8 iPSC
b
Unstained
Stained
SSC-A
FSC-A
FSC-W
SSC-W
SSEA4-PE
TRA 1-60-APC
72.1
97.7
99.9
0.028
0.016
99.9
0.090
82.8
98.0
99.4
31.9
67.9
0.26
2.42E-3
c
OCT4
SOX2
SOX17
BRACHURY
FOXA2
Expression Relative to HPRT
iPSC
Mesoderm
Endoderm

Supplementary Figure 1: ZIP8K8 iPSC Line Characterization

a. Immunostainings of pluripotency markers OCT4 and NANOG as well as OCT4 and SOX2 in undifferentiated ZIP8K8 iPSCs across N=3 colonies. Scale bar: 50 µm.

b. Flow cytometry forward (FSC) versus side scatter (SSC) density plots were first used to exclude debris and to gate for the iPSC population. Then, singlets were selected based on FSC-area versus FSC-width, and SSC-area versus SSC-width. An unstained sample was used to set the appropriate negative gates and was copied to the sample stained for SSEA4 and TRA 1-60. The frequency of cells within each selected gate is shown.

c. Quantitative PCR (qPCR) analysis of *OCT4* (N=3 replicates for each lineage), *SOX2* (N=3 replicates for iPSC and endoderm lineage, N=2 replicates for mesoderm lineage), *BRACHURY* (N=2 replicates for iPSC and mesoderm lineage, N=3 replicates for endoderm lineage), *FOXA2* (N=2 replicates for iPSC and mesoderm lineages, N=3 replicates for endoderm lineage) and *SOX17* (N=3 replicates for all lineages) transcript levels obtained for undifferentiated iPSC and following a five-day differentiation in to mesoderm and endoderm lineages using the STEMdiff™ Tri-lineage Differentiation Kit. Bars represent mean.
