# Supplementary material for: Enhanced cortical neural stem cell identity through short SMAD and WNT inhibition in human cerebral organoids facilitates emergence of outer radial glial cells
Source: Nat Cell Biol. 2022 Jun 13;24(6):981–95. doi: 10.1038/s41556-022-00929-5 (PMC9203281; doi:10.1038/s41556-022-00929-5)
Supplement: Supplementary file 2 — Reporting Summary [file 41556_2022_929_MOESM2_ESM.pdf]

nature portfolio

Corresponding author(s): Yechiel Elkabetz

Last updated by author(s): Mar 28, 2022

Reporting Summary

Nature Portfolio wishes to improve the reproducibility of the work that we publish. This form provides structure for consistency and transparency in reporting. For further information on Nature Portfolio policies, see our Editorial Policies and the Editorial Policy Checklist.

Statistics

For all statistical analyses, confirm that the following items are present in the figure legend, table legend, main text, or Methods section.

n/a	Confirmed	
☐	☒	The exact sample size (*n*) for each experimental group/condition, given as a discrete number and unit of measurement
☐	☒	A statement on whether measurements were taken from distinct samples or whether the same sample was measured repeatedly
☐	☒	The statistical test(s) used AND whether they are one- or two-sided<br>*Only common tests should be described solely by name; describe more complex techniques in the Methods section.*
☐	☒	A description of all covariates tested
☐	☒	A description of any assumptions or corrections, such as tests of normality and adjustment for multiple comparisons
☐	☒	A full description of the statistical parameters including central tendency (e.g. means) or other basic estimates (e.g. regression coefficient) AND variation (e.g. standard deviation) or associated estimates of uncertainty (e.g. confidence intervals)
☐	☒	For null hypothesis testing, the test statistic (e.g. *F*, *t*, *r*) with confidence intervals, effect sizes, degrees of freedom and *P* value noted<br>*Give P values as exact values whenever suitable.*
☐	☒	For Bayesian analysis, information on the choice of priors and Markov chain Monte Carlo settings
☐	☒	For hierarchical and complex designs, identification of the appropriate level for tests and full reporting of outcomes
☐	☒	Estimates of effect sizes (e.g. Cohen's *d*, Pearson's *r*), indicating how they were calculated

Our web collection on statistics for biologists contains articles on many of the points above.

Software and code

Policy information about availability of computer code

Data collection	DESeq2 v1.26.0; samtools v1.10; STAR v2.6.1d; HTSeq v0.10.0; sva v3.30.1; Trimmomatic v0.36; RSEM v1.3.1; Cell Ranger v3.1.0; scanpy v1.5.1; velocyto v0.17.16; scvelo v0.1.24; scrublet v0.2.3; scanorama v1.7.
Data analysis	Custom code and documentation for figure generation is available at https://github.com/daniel-rosebrock/BrainOrganoids.

For manuscripts utilizing custom algorithms or software that are central to the research but not yet described in published literature, software must be made available to editors and reviewers. We strongly encourage code deposition in a community repository (e.g. GitHub). See the Nature Portfolio guidelines for submitting code & software for further information.

Data

Policy information about availability of data

All manuscripts must include a data availability statement. This statement should provide the following information, where applicable:

- Accession codes, unique identifiers, or web links for publicly available datasets
- A description of any restrictions on data availability
- For clinical datasets or third party data, please ensure that the statement adheres to our policy

RNA sequencing datasets derived from cell lines H9, ZIP13K5 and KUCG2 have been deposited in the Gene Expression Omnibus (GEO) under the accession code of GSE189981. RNA sequencing datasets derived from cell lines ZIP8K8 and FOK1 have been deposited in the European Genome-Phenome Archive (EGA) under the accession code of EGAS00001006063. Previously published scRNA-Seq data that were re-analysed here are available under accession code GSE132672. Previously published bulk RNA-Seq data that were re-analysed here from the BrainSpan Atlas of the Developing Human Brain are available at https://www.brainspan.org/static/download.html under "RNA-Seq Gencode v10 summarized to genes". Source data are provided with this study. All other data supporting the findings of this study are available from the corresponding author on reasonable request.

Field-specific reporting

Please select the one below that is the best fit for your research. If you are not sure, read the appropriate sections before making your selection.

☒ Life sciences ☐ Behavioural & social sciences ☐ Ecological, evolutionary & environmental sciences

For a reference copy of the document with all sections, see nature.com/documents/nr-reporting-summary-flat.pdf

Life sciences study design

All studies must disclose on these points even when the disclosure is negative.

Sample size	No sample-size calculations were performed a priori. Replicates of 8 individual organoids (larger than the recommended 3 replicates for differential expression analysis) across 3 protocols for bulk RNA sequencing provided a large sample size to detect significantly up and down regulated genes across each protocol. Analyses of scRNA-seq data involved thousands of cells per organoid derivation protocol, providing a robust sample size.
Data exclusions	Common scRNA-seq quality control metrics were applied to identify true cells (exclude potential doublets and low-quality cells). The exact thresholds were determined empirically using the density of all single cells to determine appropriate, dataset-specific thresholds. Estimated doublets using scrublet v0.2.3 were removed from downstream analyses of day 50 and day 80 organoids. No bulk RNA-Seq samples were excluded. Exclusion criteria were not pre-established.
Replication	Number of independent experiments are reported in figure legends.
Randomization	All samples were randomly picked in each experiment.
Blinding	Blinding was not relevant to this study, and thus not performed.

Reporting for specific materials, systems and methods

We require information from authors about some types of materials, experimental systems and methods used in many studies. Here, indicate whether each material, system or method listed is relevant to your study. If you are not sure if a list item applies to your research, read the appropriate section before selecting a response.

Materials & experimental systems

n/a	Involved in the study
☐	☒ Antibodies
☐	☒ Eukaryotic cell lines
☒	☐ Palaeontology and archaeology
☒	☐ Animals and other organisms
☒	☐ Human research participants
☒	☐ Clinical data
☒	☐ Dual use research of concern

Methods

n/a	Involved in the study
☒	☐ ChIP-seq
☐	☒ Flow cytometry
☒	☐ MRI-based neuroimaging

Antibodies

Antibodies used

Primary Antibodies:
Rabbit Cleaved Caspase 3 (Asp 175) (Cell Signaling, #9661, 1:500)
Rabbit COUP-TF1 (Millipore, ABE1425, 1:500)
Rat CTIP2 (Abcam, ab18465, 1:250)
Mouse (IgG1) CUX1/CASP (Abcam, ab54583, 1:200)
Guinea Pig DCX (Millipore, AB2253, 1:500)
Rabbit EMX1 (Sigma-Aldrich, HPA006421, 1:50)
Rabbit EMX2 (Abcam, ab94713, 1:50)
Rabbit FOXG1 (Abcam, ab18259, 1:400)
Rabbit HOPX (Sigma-Aldrich, HPA030180, 1:500)
Mouse (IgG1) LIFR (Santa Cruz, sc-515337, 1:100)
Rabbit LMX1A (Millipore, AB10533, 1:1000)
Rabbit MEF2C (Atlas Antibodies, HPA005533, 1:100)
Mouse OCT3/4 (Santa Cruz, sc5279, 1:22)
Mouse (IgG1) OLIG3 (R&D, MAB2456, 1:450)
Mouse (IgG1) PAX6 (DSHB, supernatant, 1:22)
Rabbit PTPRZ1 (Sigma-Aldrich, HPA015103, 1:500)
Mouse (IgG1) SATB2 (Abcam, ab51502, 1:50)
Goat SOX1 (R&D, AF3369, 1:40)

Goat SOX2 (R&D, AF2018, 1:100)
Mouse SOX2 (Abcam, ab79351, 1:500)
Rabbit SP8 (Atlas Antibodies, HPA054006, 1:50)
Rabbit STMN2 (Novus Biologicals, NBP149461, 1:1000)
Rabbit TBR1 (Abcam, ab31940, 1:500)
Rabbit TCF7L2 (Cell Signaling, #2569, 1:500)
Sheep TTR (Biorad, AHP1837, 1:500)
Mouse (IgG2b) VIMENTIN (phospho S55) (Abcam, ab22651, 1:120)

Secondary Antibodies:
All secondary Antibodies were diluted 1:700

Alexa Fluor donkey anti-rabbit IgG (H+L) 488 (Invitrogen, A32790)
Alexa Fluor donkey anti-goat IgG (H+L) 546 (Invitrogen, A11056)
Alexa Fluor donkey anti-mouse IgG (H+L) 546 (Invitrogen, A10036)
Alexa Fluor goat anti-mouse IgG1 546 (Invitrogen, A21123)
Alexa Fluor goat anti-rabbit IgG (H+L) 546 (Invitrogen, A11010)
Alexa Fluor goat anti-mouse IgG2b 546 (Invitrogen, A21143)
Alexa Fluor chicken anti-rat IgG (H+L) 647 (Invitrogen, A21472)
Alexa Fluor donkey anti-mouse IgG (H+L) 647 (Invitrogen, A32787)
Alexa Fluor donkey anti-goat IgG (H+L) 647 (Invitrogen, A21447)
Alexa Fluor donkey anti-rabbit IgG (H+L) 647 (Invitrogen, A31573)
Alexa Fluor donkey anti-sheep IgG (H+L) 647 (Invitrogen, A21448)
Alexa Fluor goat anti-rabbit IgG (H+L) 647 (Invitrogen, A21244)
Alexa Fluor goat anti-mouse IgG2b 647 (Invitrogen, A21242)
Alexa Fluor goat anti-mouse IgG (H+L) 647 (Invitrogen, A21235)
Goat anti-guinea pig IgG (H+L) DyLight 650 (Abcam, ab102377)

Validation

Validation:
According to the manufacturer's website and/or CiteAb (https://www.citeab.com/antibodies):
The rabbit Cleaved Caspase 3 (Asp 175) antibody (Cell Signaling, #9661, 1:500) is reactive to human rat and mouse, and has been cited in 5672 publications.
The rabbit COUP-TF1 antibody (Millipore, ABE1425, 1:500) is reactive to human and mouse, and has been cited in 4 publications.
The rat CTIP2 antibody (Abcam, ab18465, 1:250) is reactive to human and mouse, and has been cited in 567 publications.
The mouse (IgG1) CUX1/CASP antibody (Abcam, ab54583, 1:200) is reactive to human, and has been cited in 19 publications.
The guinea Pig DCX antibody (Millipore, AB2253, 1:500) is reactive to human, rat and mouse, and has been cited in 16 publications.
The rabbit EMX1 antibody (Sigma-Aldrich, HPA006421, 1:50) is reactive to human and mouse, and has been cited in 9 publications.
The rabbit EMX2 antibody (Abcam, ab94713, 1:50) is reactive to human and mouse, and has been cited in 57 publications.
The rabbit FOXG1 antibody (Abcam, ab18259, 1:400) is reactive to human, rat and mouse, and has been cited in 88 publications.
The rabbit HOPX antibody (Sigma-Aldrich, HPA030180, 1:500) is reactive to human, and has been cited in 18 publications.
The mouse (IgG1) LIFR antibody (Santa Cruz, sc-515337, 1:100) is reactive to human, rat and mouse, and has been cited in 12 publications.
The rabbit LMX1A antibody (Millipore, AB10533, 1:1000) is reactive to human, rat, hamster and mouse, and has been cited in 46 publications.
The rabbit MEF2C antibody (Atlas Antibodies, HPA005533, 1:100) is reactive to human, and has been cited in 5 publications.
The mouse OCT3/4 antibody (Santa Cruz, sc5279, 1:22) is reactive to human, rat and mouse, and has been cited in 2,167 publications.
The mouse (IgG1) OLIG3 antibody (R&D, MAB2456, 1:450) is reactive to human and mouse, and has been cited in 3 publications.
The mouse (IgG1) PAX6 antibody (DSHB, supernatant, 1:22) is reactive to human, rat, mouse, and has been cited in 67 publications.
The rabbit PTPRZ1 antibody (Sigma-Aldrich, HPA015103, 1:500) is reactive to human, and has been cited in 16 publications.
The mouse (IgG1) SATB2 antibody (Abcam, ab51502, 1:50) is reactive to human and mouse, and has been cited in 206 publications.
The goat SOX1 antibody (R&D, AF3369, 1:40) is reactive to human, rat and mouse, and has been cited in 44 publications.
The goat SOX2 antibody (R&D, AF2018, 1:100) is reactive to human, rat and mouse, and has been cited in 130 publications.
The mouse SOX2 antibody (Abcam, ab79351, 1:500) is reactive to human and mouse, and has been cited in 52 publications.
The rabbit SP8 antibody (Atlas Antibodies, HPA054006, 1:50) is reactive to human, and has been cited in 1 publication.
The rabbit STMN2 antibody (Novus Biologicals, NBP149461, 1:1000) is reactive to human, rat and mouse, and has been cited in 41 publications.
The rabbit TBR1 antibody (Abcam, ab31940, 1:500) is reactive to human, rat, and mouse, and has been cited in 342 publications.
The rabbit TCF7L2 antibody (Cell Signaling, #2569, 1:500) is reactive to human, and has been cited in 105 publications.
The sheep TTR antibody (Biorad, AHP1837, 1:500) is reactive to human, and has been cited in 5 publications.
The mouse (IgG2b) VIMENTIN antibody (phospho S55) (Abcam, ab22651, 1:120) is reactive to human, mouse and rat, and has been cited in 44 publications.

Eukaryotic cell lines

Policy information about cell lines

Cell line source(s)

The BAC transgenic HES5::eGFP Notch activation human ES cell reporter line has been derived from the WA-09, XX (H9) human ES cell (hESC) line (Wicell). The ZIP8K8 iPSC line (ZIP gGmbH) was derived from Human dermal fibroblast (HDF) cells and obtained from Franz-Josef Müller at University Hospital Schleswig Holstein, Kiel. The human fibroblast derived iPSC line ZIP13K5 was obtained from Franz-Josef Müller at University Hospital Schleswig Holstein, Kiel. The human PBMC derived iPSC line FOK1 was received from Michael Ziller from the Max Planck Institute for Psychiatry in Munich. The human fibroblast derived iPSC line KUCG2 was ordered from EBiSC.

Authentication

The pluripotency of ZIP8K8 iPSC line was assessed by a five-day differentiation in to mesoderm and endoderm lineages using

Authentication	the STEMdiff™ Tri-lineage Differentiation Kit, followed by qPCR analysis of OCT4, SOX2, BRACHURY, FOXA2 and SOX17 transcript levels. Additionally, all cell lines were obtained using MTA approval, and were karyotyped at the time of generation.
Mycoplasma contamination	Negative for mycoplasma infection
Commonly misidentified lines (See ICLAC register)	No misidentified line has been used

Flow Cytometry

Plots

Confirm that:

☒ The axis labels state the marker and fluorochrome used (e.g. CD4-FITC).

☒ The axis scales are clearly visible. Include numbers along axes only for bottom left plot of group (a 'group' is an analysis of identical markers).

☒ All plots are contour plots with outliers or pseudocolor plots.

☒ A numerical value for number of cells or percentage (with statistics) is provided.

Methodology

Sample preparation	Single cells were generated by treating the hiPSC line with accutase enzyme, then the cells were blocked using blocking reagent (similar reagent used in immunofluroscence staining), then cell suspension was incubated with respective antibody (concentration were used as mentioned by the provider) and the cell suspension was analyzed with respectively unstained population.
Instrument	FACS Aria II
Software	Flow jo
Cell population abundance	No cell sorting was performed.
Gating strategy	Cells were first gated by Forward and Side Scatter to exclude debris and doublets. Then, target cell population were gated by SSEA4 and TRA 1-60. Isotype controls were used to define background and non-specific signal.

☒ Tick this box to confirm that a figure exemplifying the gating strategy is provided in the Supplementary Information.
